# Thermodynamic and Molecular Characterization of Adsorption on Zeolites: A Unified Framework Combining Inverse Gas Chromatography, Hamaker Theory, and Nonlinear Lewis Acid–Base Modeling

**DOI:** 10.3390/molecules31101760

**Published:** 2026-05-20

**Authors:** Tayssir Hamieh, Mouhamad Rachini, Soumaya Hamieh, Mohammad Mahdi Assaf, Zeinab Hamie, Khaled Chawraba, Thibault Roques-Carmes, Joumana Toufaily

**Affiliations:** 1 Faculty of Science and Engineering, Maastricht University, P.O. Box 616, 6200 MD Maastricht, The Netherlands; 2 Institut de Science des Matériaux de Mulhouse, Université de Haute-Alsace, CNRS, IS2M UMR 7361, 68100 Mulhouse, France; 3 Laboratory of Materials, Catalysis, Environment and Analytical Methods Laboratory (MCEMA), and LEDDAR Laboratory, Lebanese University, Hadath, Beirut P.O. Box 6573/14, Lebanon; mouhamadrachini@outlook.com (M.R.); soumaya.hamieh@hotmail.fr (S.H.); mohammadmahdiassaf@gmail.com (M.M.A.); zaynabahdhamei@gmail.com (Z.H.); khaled.chawraba@outlook.com (K.C.); joumana.toufaily@ul.edu.lb (J.T.); 4 Laboratoire Réactions et Génie des Procédés, UMR 7274 CNRS, Université de Lorraine, 54000 Nancy, France; thibault.roques-carmes@univ-lorraine.fr; 5 TIMR (Integrated Transformations of Renewable Matter), Royallieu Research Center, University of Technology de Compiegne, ESCOM, CS 60 319, CEDEX, 60203 Compiegne, France; 6 Department of Civil and Environmental Engineering, The Hong Kong Polytechnic University, ZS972, Hung Hom, Kowloon, Hong Kong

**Keywords:** surface energy, Lewis parameters, Hamaker constant, surface area, thermodynamics, adsorption, porous materials

## Abstract

A comprehensive thermodynamic and molecular-level investigation of adsorption on MgY and NH_4_Y zeolites is presented using inverse gas chromatography at infinite dilution (IGC-ID), combined with a Hamaker-based formalism and an extended five-parameter Lewis acid–base model. The study introduces a unified framework that integrates dispersive, polar, and donor–acceptor interactions while explicitly accounting for temperature-dependent intermolecular geometry. The results demonstrate that the London dispersive free energy exhibits a highly linear temperature dependence (R^2^ > 0.999), while the corresponding surface energy decreases linearly with temperature (e.g., $\gamma_{s}^{d}\left(T\right)=-0.297T+189.48$ mJ·m^−2^ for MgY), reflecting the progressive weakening of dispersion forces. Simultaneously, the intermolecular separation distance follows a linear relation $r(T)=r_{0}+\alpha_{\mathrm{eff}}T$, with $\alpha_{\mathrm{eff}}$ values on the order of (2–3) × 10^−3^ Å·K^−1^ for MgY, enabling the determination of intrinsic contact distances $r_{0}$ at 0 K, varying between 4.00 Å and 6.60 Å. A major finding is that the molecular surface area of adsorbed probes is not constant but follows a quadratic temperature dependence with excellent accuracy (R^2^ > 0.999), establishing adsorption cross-section as a thermodynamic variable. The comparison between MgY and NH_4_Y reveals two distinct adsorption regimes: MgY exhibits a structured and strongly dispersive interaction field associated with Mg^2+^ cations, whereas NH_4_Y displays enhanced polarity, stronger specific interactions, and greater molecular flexibility driven by hydrogen bonding and protonic effects. Thermodynamic analysis of Lewis acid–base interactions shows that classical linear models are insufficient. Statistical evaluation (R^2^ ≈ 0.986, minimum AIC/BIC, lowest RMSE) demonstrates that the five-parameter Hamieh model provides the most accurate and physically meaningful description, capturing nonlinear donor–acceptor interactions and amphoteric coupling effects. Overall, this work establishes a novel thermodynamic methodology that quantitatively links macroscopic surface energetics to microscopic interaction parameters, providing new insight into adsorption mechanisms and a robust framework for the rational design of porous materials in catalysis, separation, and energy applications.

## 1. Introduction

Understanding the physicochemical properties of solid surfaces remains a central challenge in surface and materials science, particularly for porous systems such as zeolites, where adsorption phenomena arise from a complex interplay between dispersive, electrostatic, and acid–base interactions. Among these materials, FAU-type zeolites, and especially Y zeolites, occupy a prominent position owing to their high specific surface area, well-defined pore architecture, and remarkable framework tunability [1,2,3]. The possibility of cation exchange within the aluminosilicate lattice provides a powerful means of tailoring surface energetics, enabling precise control over adsorption behavior and catalytic performance [2,3].

Within this family, MgY and NH_4_Y zeolites represent two fundamentally distinct physicochemical systems. MgY is characterized by the presence of divalent Mg^2+^ cations, which generate strong local electrostatic fields and act as pronounced Lewis acid sites capable of inducing significant polarization in adsorbed molecules [4,5]. In contrast, NH_4_Y is dominated by ammonium species, which behave as precursors of Brønsted acidity and are associated with a softer and more deformable electronic environment [6]. These differences lead to markedly distinct adsorption mechanisms, reflecting the balance between dispersion forces, electron donor–acceptor interactions, and induction effects.

These distinctions are particularly evident in catalytic applications, where Y zeolites play a key role in industrial processes [2,7]. The ammonium form, upon thermal activation, generates protonic sites responsible for acid-catalyzed reactions such as fluid catalytic cracking, hydrocarbon isomerization, and alkylation [7,8,9]. In contrast, the introduction of Mg^2+^ cations promotes strong Lewis acidity, favoring reactions involving electron donor molecules, including alcohol dehydration, carbonyl activation, and CO_2_ conversion [4,10,11]. The coexistence of Lewis and Brønsted acid sites may also result in bifunctional catalytic behavior, enhancing activity and selectivity through synergistic effects [5,10].

Beyond catalysis, FAU-type zeolites are widely employed in adsorption and separation processes, where their large supercages and interconnected pore networks enable selective interactions with a broad range of molecules [12,13,14]. NH_4_Y exhibits strong affinity toward polar and hydrogen-bonding species, making it particularly effective for the removal of water, alcohols, and other polar compounds [13,15]. In contrast, MgY preferentially interacts with electron donor molecules due to its Lewis acidic centers, making it suitable for applications such as CO_2_ capture, gas purification, and selective adsorption of heteroatom-containing compounds [4,16,17,18].

At a more fundamental level, these behaviors originate from the electronic structure of the zeolite surface. Dispersive interactions, arising from London forces, depend on deformation polarizability and ionization energy, whereas electrostatic and induction contributions are strongly influenced by the nature of extra-framework cations [19,20,21,22,23,24]. MgY, with its high charge density, enhances polarization and induction effects, whereas NH_4_Y exhibits greater electronic flexibility and stronger contributions from dispersion and hydrogen bonding.

Despite the extensive use of Y zeolites, a unified and quantitative description of their surface properties remains challenging. Conventional approaches often treat dispersive and acid–base interactions separately and rely on simplified assumptions that fail to capture the complexity of real systems [12,22]. In particular, classical inverse gas chromatography (IGC) methods—such as those of Dorris–Gray and Fowkes [23,24,25,26,27,28,29,30,31,32,33,34,35,36,37]—assume fixed intermolecular distances and temperature-invariant interaction fields, neglecting the intrinsic coupling between adsorption energy, molecular separation, and thermal effects.

This limitation is evident in earlier studies on zeolites, including the work of Bilgiç and Tümsek [38] and related contributions from Tümsek, İnel, and co-workers [39,40], which rely on classical IGC formalisms. While these studies provide useful insights, they do not explicitly account for the temperature dependence of intermolecular distances, surface heterogeneity, or nonlinear interaction effects. As a result, such approaches may lead to significant uncertainties in the determination of surface energetics and acid–base parameters. Furthermore, they do not adequately describe the complex host–guest interactions and confinement effects inherent to microporous materials.

Although inverse gas chromatography has been extensively applied for the determination of surface properties over the past decades, most existing methodologies remain based on classical formalisms developed several decades ago, with limited evolution in their fundamental thermodynamic treatment. Recent advances have begun to address these limitations by introducing temperature-dependent and physically consistent descriptions of adsorption, highlighting the need for a renewed theoretical framework capable of capturing the true molecular complexity of surface interactions.

To overcome these limitations, a comprehensive thermodynamic framework has recently been developed [41,42,43,44,45,46,47,48,49,50,51], demonstrating the strong temperature dependence of molecular surface area [41,42,43,44,45] and introducing an extended five-parameter model for Lewis acid–base interactions [46,47,48,49,50,51]. This approach enables a unified description of adsorption energetics, incorporating nonlinear donor–acceptor interactions, amphoteric coupling, and higher-order effects. It has been validated across a wide range of materials, including polymers, metal oxides, carbon nanomaterials, and zeolites, showing superior predictive capability compared to classical models.

In addition, recent developments have established a direct relationship between dispersive adsorption energy and fundamental electronic properties through a London-based formulation involving deformation polarizability, ionization energy, and intermolecular distance [46,47,48,49,50,51]. Within this framework, dispersive surface energy becomes an intrinsic thermodynamic quantity that can be rigorously determined and experimentally validated. These advances highlight the necessity of integrating electronic structure, thermodynamics, and molecular geometry into a consistent description of adsorption.

Despite these significant developments, a detailed and unified thermodynamic characterization of cation-exchanged Y zeolites, particularly MgY and NH_4_Y, remains lacking. These systems provide an ideal platform for investigating the transition between two distinct regimes of surface behavior: a highly structured, electrostatically dominated surface (MgY) and a more flexible, hydrogen-bonding-driven surface (NH_4_Y). Such a comparative study offers valuable insight into the coupling between electronic structure, surface heterogeneity, and adsorption thermodynamics.

In this work, a comprehensive investigation of MgY and NH_4_Y zeolites is carried out using inverse gas chromatography at infinite dilution, combined with the advanced thermodynamic framework described above. The objectives are to:

- Determine the London dispersive surface energy as a function of temperature using a rigorous electronic formulation;
- Quantify the Lewis acid–base properties using the five-parameter model, including nonlinear contributions;
- Establish a unified description of adsorption by correlating thermodynamic data with fundamental molecular parameters such as ionization energy, deformation polarizability, and intermolecular distance.

This approach provides a physically consistent and predictive framework for understanding adsorption phenomena at the molecular level and contributes to the rational design of porous materials for catalysis, separation, and energy-related applications.

## 2. Theoretical Framework

A rigorous and predictive description of adsorption phenomena at solid surfaces requires a thermodynamic framework capable of capturing the full complexity of intermolecular interactions governing probe–surface affinity. In porous materials such as zeolites, these interactions originate from a subtle coupling between London dispersion forces, electrostatic (induction) effects, and Lewis acid–base interactions, all of which are strongly influenced by the electronic structure of both the adsorbate and the solid surface. Despite decades of investigation, most conventional approaches treat these contributions separately and often rely on simplified or semi-empirical assumptions, which limit their predictive capability, particularly for heterogeneous and amphoteric surfaces.

In this context, inverse gas chromatography at infinite dilution (IGC-ID) [25,26,27,28,29,30,31,38,39,40,41,42,43,44,52,53,54,55,56,57,58,59,60,61,62,63,64,65] offers a unique experimental route to probe surface thermodynamics at the molecular scale. By operating in the limit of vanishing probe coverage, IGC-ID eliminates lateral interactions between adsorbed molecules, ensuring that the measured retention behavior reflects exclusively the intrinsic interaction between an isolated probe molecule and the surface. This feature provides direct access to the standard free energy of adsorption, which constitutes the fundamental thermodynamic quantity governing surface interactions.

The net retention volume $V_{N}$ of solvents was determined using the IGC technique at infinite dilution, leading to the Gibbs free energy $-∆G_{a}^{0}(T)$ of organic solvents adsorbed on the solids through Equation (1):

(1) $$∆G_{a}^{0}(T)=-\;RT\;ln\;\left(\frac{V_{n}P_{0}}{Sm\pi_{0}}\right)$$

where m is the mass of the solid, S its specific surface area, and $P_{0}$ and $\pi_{0}$ are respectively the standard pressure and the two-dimensional pressure determined by one of the two reference states of Kemball and Rideal [38] or De Boer and Kruyer [39].

The key advantage of this formulation lies in its generality: it enables the systematic decomposition of the adsorption free energy into distinct physical contributions, elucidated by Equation (2):

(2) $$\Delta G_{a}^{0}=\Delta G_{a}^{d}+\Delta G_{a}^{p}$$

where $\Delta G_{a}^{d}$ represents the dispersive (London) component and $\Delta G_{a}^{p}$ accounts for polar interactions, including Lewis acid–base and polar effects. For nonpolar probes such as n-alkanes, the adsorption free energy reduces to the dispersive contribution, providing a direct and unambiguous access to the London interaction energy.

### 2.1. London Dispersive and Polar Interactions: A Molecular Electronic Approach

A central novelty of the present methodology lies in the explicit formulation of dispersive interactions in terms of fundamental electronic properties, moving beyond traditional geometric or empirical treatments. In contrast to classical methods (e.g., Dorris–Gray or Schultz approaches), which approximate dispersive interactions using incremental surface energies, the present approach is grounded in a rigorous London theory, where the adsorption energy is expressed by Equation (3) as a function of deformation polarizability, ionization energy, and intermolecular separation.

(3) $$\Delta G_{a}^{d}=-\frac{3N\alpha_{0,S}\alpha_{0,X}}{2(4\pi \varepsilon_{0}{)}^{2}r_{S-X}^{6}}\cdot \frac{\varepsilon_{S}\varepsilon_{X}}{\varepsilon_{S}+\varepsilon_{X}}$$

where N is Avogadro’s number, $\varepsilon_{0}$ the permittivity of vacuum, *S* denoting the solid particle and *X* the solvent molecule separated by a distance $r_{S-X}$, $\alpha_{0}\;$ the deformation polarizability, and ε the ionization energy.

For **nonpolar probes**, such as n-alkanes, the adsorption process is governed exclusively by **London dispersive interactions**, since their polar contribution is negligible. Consequently, the dispersive component of the free energy of adsorption is equal to the total free energy: $\left(-∆G_{a}^{d}\right)(n-alkanes)=\left(-∆G_{a}^{0}\right)\;(n-alkanes)$.

In contrast, for a **polar probe** X, the total free energy of adsorption includes both dispersive and specific (polar) contributions. The polar component $\left(-\Delta G_{a}^{p})(X\right)$ is therefore obtained by subtracting the dispersive contribution from the total adsorption free energy.

The dispersive parameter $P_{S\left(n-alkane\right)}$ of n-alkanes is defined by Equation (4):

(4) $$P_{S\left(n-alkane\right)}=\frac{\varepsilon_{S}\;\varepsilon_{\left(n-alkane\right)}}{\left(\varepsilon_{S}+\;\varepsilon_{\left(n-alkane\right)}\right)}\alpha_{0(n-alkane)}$$

The linear relationship between the dispersive free energy and this parameter is expressed by Equation (5) as:

(5) $$\left(-∆G_{a}^{0}\right)\left(T\right)=A\;P_{S\left(n-alkane\right)}+C$$

Using this correlation, the reference dispersive contribution for the polar probe X is given by $AP_{SX}+C$, allowing from Equation (6) the polar component of the free energy to be written as:

(6) $$\left(-∆G_{a}^{p}\right)\left(X\right)=\left(-∆G_{a}^{0}\right)\left(X\right)-(A\;P_{SX}+C)$$

Finally, the temperature dependence of the polar free energy enables the determination of the corresponding polar enthalpy $\left(-\Delta H_{a}^{p})(X\right)$ and polar entropy $\left(-\Delta S_{a}^{p})(X\right)$, according to Equation (7):

(7) $$\left(-∆G_{a}^{p}\right)\left(X,\;T\right)=\left(-∆H_{a}^{p}\right)\left(X\right)-T\;\left(-∆S_{a}^{sp}\right)\left(X\right)$$

This formulation highlights a fundamental aspect often overlooked in conventional analyses: London dispersion forces are intrinsically electronic in nature, and their magnitude is controlled by:

- the deformation polarizability of both interacting media,
- their ionization energies,
- and the equilibrium intermolecular distance.

An important consequence of this expression is that dispersive interactions are not merely additive or size-dependent, but are governed by the electronic response of the system, which provides a direct link between macroscopic adsorption measurements and microscopic electronic properties.

Within the present framework, the experimental determination of the dispersive component of the adsorption free energy enables direct access to fundamental intermolecular parameters. In particular, the center-to-center distance between the adsorbate molecule and the solid surface, $r_{X-S}(T)$, can be derived as a function of temperature by inverting the London interaction expression. This leads to Equation (8):

(8) $$r_{X-S}\left(T\right)={\left[\frac{3N}{{2\left(4\pi \varepsilon_{0}\right)}^{2}}\left[\left(\frac{\varepsilon_{S}\;\varepsilon_{X}}{\left(\varepsilon_{S}+\;\varepsilon_{X}\right)}\alpha_{S}\alpha_{X}\right)\right]\frac{1\;}{\left(-∆G_{a}^{d}\left(T\right)\right)\;}\right]}^{1/6}$$

From this intermolecular distance, the equilibrium separation distance or London potential minimum distance, denoted $D_{0}(T)$, is defined by Equation (9):

(9) $$D_{0}\left(T\right)=\frac{r_{X-S}\left(T\right)}{2}$$

This characteristic distance plays a central role in the evaluation of surface energetic properties. In particular, the London dispersive surface energy of the solid, $\gamma_{s}^{d}(T)$, is determined at each temperature by combining the experimentally derived parameters with the Hamaker formalism in Equation (10):

(10) $$\gamma_{s}^{d}\left(T\right)={\left(\sqrt{\gamma_{l}^{d}\left(T\right)}+\sqrt{\frac{A_{12}\left(T\right)}{24\pi {D_{0}\left(T\right)}^{2}}}\right)}^{2}$$

where $A_{12}(T)$ represents the Hamaker constant for the interaction between the solid and the probe molecule, and $\gamma_{l}^{d}(T)$ is the dispersive surface energy of the liquid probe.

Furthermore, the effective molecular surface area of the adsorbed probes, whether nonpolar or polar, can be evaluated as a function of temperature using the Fowkes approach [27]. By combining the dispersive free energy with the corresponding surface energies of the solid and the probe, one obtains Relation (11):

(11) $$a\left(T\right)=\frac{\left(-∆G_{a}^{d}\left(T\right)\right)}{2N\sqrt{\left(\gamma_{l}^{d}(T)\gamma_{s}^{d}(T)\right)}}$$

In this context, a key feature of the present methodology lies in the explicit treatment of the intermolecular distance r(T) given by Equation (12) as a temperature-dependent quantity, rather than a constant parameter:

(12) $$r(T)=r_{0}+\alpha T,$$

where $r_{0}$ represents the equilibrium distance at 0 K and α is an effective thermal expansion coefficient. This leads to a natural description of the temperature dependence of the dispersive interaction, thereby allowing the extraction of intrinsic structural and thermodynamic parameters from experimental IGC data.

### 2.2. London Dispersive Surface Energy and Hamaker-Based Description

Another major advancement of this methodology is the integration of Lifshitz theory and Hamaker constants into the interpretation of IGC measurements given by Equation (10). Rather than treating the dispersive surface energy $\gamma_{s}^{d}$ as an empirical parameter, it is here derived from first principles through its relationship with the Hamaker constant $A_{12}$, which encapsulates the collective effect of electromagnetic fluctuations between interacting media.

Within this framework, the dispersive surface energy is expressed as a function of:

- The static dielectric permittivity ε(0);
- The refractive index n;
- The characteristic electronic transition frequency $\nu_{e}$, thereby linking macroscopic surface properties to fundamental optical and electronic parameters.

A key outcome of this approach is the recognition that the **high-frequency (UV) contribution dominates the Hamaker constant**, implying that dispersive interactions are primarily controlled by electronic transitions rather than thermal fluctuations. This provides a robust explanation for the relatively weak temperature dependence of dispersive surface energies and highlights the dominant role of the substrate electronic structure.

### 2.3. Lewis Acid–Base Interactions: Beyond Linear Models

While dispersive interactions govern the behavior of nonpolar probes, the adsorption of polar molecules requires a detailed description of **Lewis acid–base interactions**, which are inherently more complex. Classical approaches based on Gutmann donor and acceptor numbers provide a useful starting point but are fundamentally limited by their linear nature and inability to capture nonlinear and cooperative effects.

To address these limitations, the present work employs a **five-parameter thermodynamic model**, which constitutes a significant conceptual and methodological advancement. The polar component of the adsorption free energy is expressed by Equation (13):

(13) $$\Delta G_{a}^{p}=-\left(K_{A}AN+K_{D}DN+K\;AN\cdot DN+K_{2A}AN^{2}+K_{2D}DN^{2}\right)$$

where AN and DN are respectively the corrected acceptor number and donor number of the adsorbed solvents, $K_{A}$ is the Lewis acidity parameter traducing the ability of the surface to accept electrons (Lewis acid sites), $K_{D}$ is the Lewis basicity parameter representing the ability to donate electrons (basic sites), K is an interaction, an amphoteric coupling between acid and base interactions, and $K_{2A},K_{2D}$ are the nonlinear acid–base contributions traducing the higher-order polarization effects.

This model is particularly well-suited for zeolites, and introduces higher-order terms and coupling parameters that account for amphoteric behavior, nonlinear polarization effects, and interaction synergy between donor and acceptor contributions.

From a physical standpoint, the parameters $K_{A}$ and $K_{D}$ quantify the intrinsic Lewis acidity and basicity of the surface, while the additional terms K, $K_{2A}$, and $K_{2D}$ capture deviations from ideal linearity, which are particularly pronounced in systems exhibiting strong electrostatic fields or heterogeneous active sites, such as zeolites.

### 2.4. Coupled Nature of Surface Interactions: A Unified Perspective

A key conceptual strength of the present methodology lies in the recognition that **dispersive and acid–base interactions are intrinsically coupled**, rather than independent contributions. In particular, strong electrostatic fields, such as those generated by divalent cations in MgY zeolites, enhance molecular polarization and thereby modify both dispersive and specific interactions. Conversely, highly polarizable systems, such as NH_4_Y, exhibit enhanced London interactions due to their softer electronic structure.

This coupling is naturally captured within the present framework, where the total free energy of adsorption given by Equation (2), emerges as a unified thermodynamic quantity reflecting the global interaction between the probe molecule and the surface. Such an approach provides a more realistic and physically meaningful description of adsorption phenomena, particularly for complex and heterogeneous materials.

### 2.5. Relevance to MgY and NH_4_Y Zeolites

The application of this advanced IGC-based methodology to MgY and NH_4_Y zeolites offers a unique opportunity to investigate the Lewis acid–base surface behavior of MgY and NH_4_Y zeolites, two systems where electrostatic and induction effects dominate, leading to significant nonlinear contributions to adsorption.

This behavior makes these two materials particularly well-suited for validating the present theoretical framework and for demonstrating its ability to capture the transition between **dispersion-dominated and electrostatically dominated adsorption regimes**. More broadly, it highlights the potential of this methodology as a **general predictive tool** for the characterization of surface properties across a wide range of materials.

## 3. Experimental Section

### 3.1. Materials

Two FAU-type zeolites were investigated in this study: NH_4_Y (ammonium Y zeolite) and MgY (magnesium-exchanged Y zeolite). The parent NaY zeolite was supplied in powder form from Aldrich (Paris, France).

The MgY form was prepared by ion exchange of the parent NaY zeolite using a 0.53 mol·L^−1^ Mg(NO_3_)_2_ aqueous solution under controlled conditions (96 h contact time, liquid-to-solid ratio of 20 cm^3^·g^−1^, room temperature), followed by filtration, extensive washing to remove residual nitrate and sodium ions, and drying under controlled conditions to ensure stabilization of the exchanged material.

The MgY zeolite was prepared via ion exchange of the parent Y zeolite using an aqueous magnesium salt solution (typically MgCl_2_), ensuring a high degree of exchange of extra-framework cations. After exchange, the sample was thoroughly washed with deionized water to remove residual ions and subsequently dried at moderate temperature (typically 373–423 K) to eliminate physisorbed water without altering the framework structure.

Prior to chromatographic measurements, both samples were subjected to thermal pre-treatment under inert gas flow (helium) at temperatures between 423 K and 573 K, in order to remove moisture and volatile impurities while preserving the structural integrity of the zeolite. The samples were then cooled to the measurement temperature under helium flow to avoid readsorption of atmospheric species. The probe molecules used in this study included:

- Nonpolar probes: n-alkanes (n-pentane to n-nonane), used for dispersive interaction analysis;
- Polar probes: dichloromethane, chloroform, acetone, ethyl acetate, tetrahydrofuran, acetonitrile, methanol, ethanol, trichloroethylene, tetrachloroethylene, diethyl ether and benzene, and cyclohexane, selected to cover a wide range of donor (*DN*) and acceptor (*AN*) numbers.

All probe molecules were of high purity (≥99%) and used without further purification.

### 3.2. Column Preparation

The chromatographic columns were prepared by packing the zeolite powders into stainless steel columns (typically 30 cm in length, 4 mm internal diameter). The packing was performed carefully to ensure homogeneous distribution of the solid, minimal channeling effects, and reproducible flow conditions.

The mass of the packed solid was determined with high precision (±0.1 mg), as it directly affects the calculation of the net retention volume.

### 3.3. Inverse Gas Chromatography Measurements

Inverse gas chromatography experiments were carried out using a gas chromatograph equipped with a flame ionization detector (FID), a high-precision temperature control system (±0.1 K), and a constant flow controller for the carrier gas.

Helium (purity ≥ 99.999%) was used as the carrier gas. The flow rate was carefully regulated and measured using a calibrated flowmeter, with typical values in the range of 20–30 mL·min^−1^.

Measurements were performed in the temperature range 300–400 K, ensuring operation in the infinite dilution regime, where probe injections were sufficiently small to avoid surface saturation and lateral interactions.

Each probe molecule was injected using a microsyringe in trace amounts (typically <0.1 µL), and the retention time $t_{R}$ was determined from the chromatographic peak maximum. The dead time $t_{0}$ was measured using methane as a non-retained probe.

The net retention volume $V_{N}$ was calculated according to Equation (14):

(14) $$V_{N}=\frac{(t_{R}-t_{0})\;F\;j}{m}$$

where F is the carrier gas flow rate corrected to standard conditions, j is the James–Martin correction factor, and m is the mass of the adsorbent.

### 3.4. Uncertainty Analysis on Retention Times

A careful analysis of experimental uncertainty is essential for ensuring the reliability of thermodynamic parameters derived from IGC measurements. In the present work, particular attention was devoted to the determination of retention times, which constitute the primary experimental observable.

Each retention time $t_{R}$ was measured at least three times, and the reported value corresponds to the average of independent injections. The associated uncertainty $\sigma_{t_{R}}$ was evaluated using the standard deviation formula, while the relative uncertainty on retention time was found to be typically:

$$\frac{\sigma_{t_{R}}}{t_{R}}\leq 0.5\%\;\mathrm{to}\;1\%$$

depending on the probe and temperature.

Additional sources of uncertainty include: dead time determination ($t_{0}$), typically within ±0.5% and flow rate measurement, with an uncertainty of ±1%.

The propagation of uncertainty from retention time to the net retention volume $V_{N}$ and subsequently to the free energy of adsorption $\Delta G_{a}^{0}$ was evaluated using standard error propagation rules. The resulting uncertainty on $\Delta G_{a}^{0}$ is estimated to be within: ±(1–2)%.

### 3.5. Reproducibility and Reliability

To ensure reproducibility, measurements were repeated at multiple temperatures, multiple probes were used for each interaction type, and consistency checks were performed on homologous series (n-alkanes).

The linearity of $\Delta G_{a}^{d}$ versus carbon number for n-alkanes confirmed the validity of the infinite dilution regime and the absence of experimental artifacts.

### 3.6. Data Analysis and Computational Methods

All thermodynamic calculations and model implementations were performed using Wolfram Mathematica (version 14.3), which enabled both symbolic and numerical treatment of the adsorption equations.

Regression analyses of experimental data, including linear and nonlinear fitting procedures, were carried out using the Excel program based on least-squares minimization. The thermodynamic parameters (ΔH, ΔS, ΔG) were obtained from the temperature dependence of adsorption free energy.

The quality of the fits was evaluated using the coefficient of determination (R^2^), root mean square error (RMSE), Akaike information criterion (AIC), and Bayesian information criterion (BIC). The high values of R^2^ and the consistency of the statistical indicators confirm the robustness and reproducibility of the analysis.

### 3.7. Structural and Surface Characteristics of MgY and NH_4_Y Zeolites

To establish a direct link between the thermodynamic analysis and the physicochemical properties of the investigated materials, it is essential to consider the structural and surface characteristics of MgY and NH_4_Y zeolites.

Both materials belong to the FAU-type zeolite family, characterized by a three-dimensional microporous framework composed of large supercages (~1.3 nm) interconnected through 12-membered oxygen rings (~0.74 nm aperture) [1,2,3]. This architecture provides a high specific surface area and facilitates the adsorption of a wide range of molecules under confinement conditions.

The key difference between the two materials arises from the nature of their extra-framework cations. In MgY, divalent Mg^2+^ cations are introduced through ion exchange, leading to a high local charge density within the pores. These cations act as strong Lewis acid sites and generate intense electrostatic fields capable of polarizing adsorbed molecules [4,5]. Such polarization enhances induction and dispersion interactions, resulting in stronger adsorption energies.

In contrast, NH_4_Y contains ammonium ions, which act as precursors of Brønsted acid sites. The NH_4_^+^ species are associated with a more diffuse charge distribution and a softer electronic environment compared to Mg^2+^. This leads to weaker electrostatic fields but facilitates hydrogen bonding and proton-donor interactions with polar molecules [6,13,15]. Upon thermal treatment, NH_4_Y may also partially transform into the protonic HY form, further increasing its Brønsted acidity [7,8,9].

These fundamental differences in surface chemistry directly influence the adsorption behavior observed in the present study. The higher dispersive adsorption energies measured for MgY are consistent with its stronger polarization capability and higher effective surface polarizability, as predicted by London dispersion theory [22,23,24]. Conversely, the lower dispersive energies and more complex thermodynamic behavior observed for NH_4_Y reflect the increasing contribution of specific interactions, particularly hydrogen bonding and proton-mediated adsorption.

Furthermore, the heterogeneity observed in the thermodynamic parameters for NH_4_Y, including the dispersion of compensation temperatures and the occurrence of non-classical behaviors, can be attributed to the coexistence of multiple adsorption sites with different energetic characteristics. Such heterogeneity has been widely reported for ammonium and protonic zeolites, where adsorption involves a combination of dispersive, electrostatic, and specific interactions within a confined microporous environment [12,13,14].

The trends observed in the present work are in good agreement with previous studies on zeolitic materials, which have shown that cation type and charge density play a dominant role in determining adsorption energetics and selectivity [4,10,16,17,18]. However, unlike classical approaches, the present methodology provides a quantitative and thermodynamically consistent framework that directly links these material properties to measurable adsorption parameters, including dispersive energy, entropy, intermolecular distance, and molecular surface area.

This explicit correlation between material characteristics and adsorption thermodynamics reinforces the physical validity of the present approach and eliminates the need for speculative assumptions. It also demonstrates that the observed differences between MgY and NH_4_Y are not empirical but originate from well-defined electronic and structural properties of the zeolite framework.

## 4. Results and Discussion

The term *molecular characterization of adsorption* used in this work is justified by the fact that the analysis goes beyond the determination of macroscopic thermodynamic quantities and provides direct access to parameters that describe adsorption at the molecular scale. In particular, the present methodology enables the determination of the temperature-dependent intermolecular separation distance r(T), the effective molecular surface area a(T), and interaction parameters derived from London dispersion theory and Lewis acid–base modeling. These quantities reflect the spatial organization, interaction strength, and electronic coupling between adsorbate molecules and the surface. Unlike conventional IGC approaches, which typically yield only global surface energy values, the present framework establishes a direct link between measurable thermodynamic data and microscopic descriptors of adsorption. In this sense, the methodology provides a genuine molecular-level characterization of adsorption processes, as it quantitatively captures the evolution of intermolecular geometry and interaction mechanisms as a function of temperature and surface chemistry.

The proposed methodology was applied to the chromatographic data in order to evaluate the London dispersive $\Delta G_{a}^{d}(T)$, and polar $\Delta G_{a}^{p}(T)$ free energy of adsorption, for the investigated solvents on MgY and NH_4_Y zeolites as a function of temperature. The resulting values are reported in Tables S1 and S2.

### 4.1. Separation Distance $r_{X-S}\left(T\right)$ Versus the Temperature

These results allowed for the separation distance $r_{S-X}\left(T\right)$ between the adsorbed solvents and MgY and NH_4_Y zeolites as a function of temperature. The values of $r_{S-X}\left(T\right)$ reported in Table S3 are used to determine the molecular surface area of adsorbed molecules and the London dispersive surface energy of the investigated zeolites as a function of temperature using the new Hamaker constant methodology.

### 4.2. Thermodynamic Parameters of London Dispersive Adsorption

The thermodynamic parameters associated with the London dispersive adsorption of probe molecules on MgY and NH_4_Y zeolites were determined from the temperature dependence of the dispersive free energy of adsorption obtained by inverse gas chromatography at infinite dilution. This approach provides direct access to the energetic and entropic contributions governing probe–surface interactions under conditions where lateral interactions are negligible.

The dispersive enthalpy $\Delta H_{a}^{d}$ and entropy $\Delta S_{a}^{d}$ of adsorption were derived from the linear variation in the dispersive free energy with temperature, expressed as:

(15) $$\Delta G_{a}^{d}\left(T\right)=\Delta H_{a}^{d}-T\Delta S_{a}^{d}$$

For each solvent–solid system, linear regression analysis was applied to the experimental $\Delta G_{a}^{d}(T)$ data, allowing accurate determination of the thermodynamic parameters corresponding to the dispersive contribution. The results, including the values of $\Delta H_{a}^{d}$, $\Delta S_{a}^{d}$, the coefficient of determination $R^{2}$, and the thermodynamic compensation temperature $T_{\mathrm{int}}$, are summarized in Table S4.

The compensation temperature $T_{\mathrm{int}}$ is defined by Equation (16):

(16) $$T_{\mathrm{int}}=\frac{\Delta H_{a}^{d}}{\Delta S_{a}^{d}}$$

and represents the characteristic temperature at which the enthalpic and entropic contributions to adsorption exactly compensate each other. When $T_{\mathrm{int}}$ falls outside the investigated temperature range (313–383 K), it should be interpreted as an extrapolated parameter reflecting the intrinsic thermodynamic behavior of the system.

**Figure 1:**
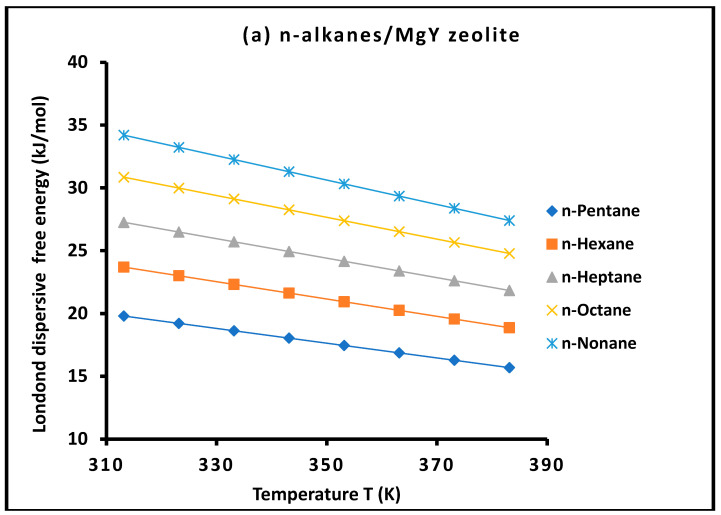

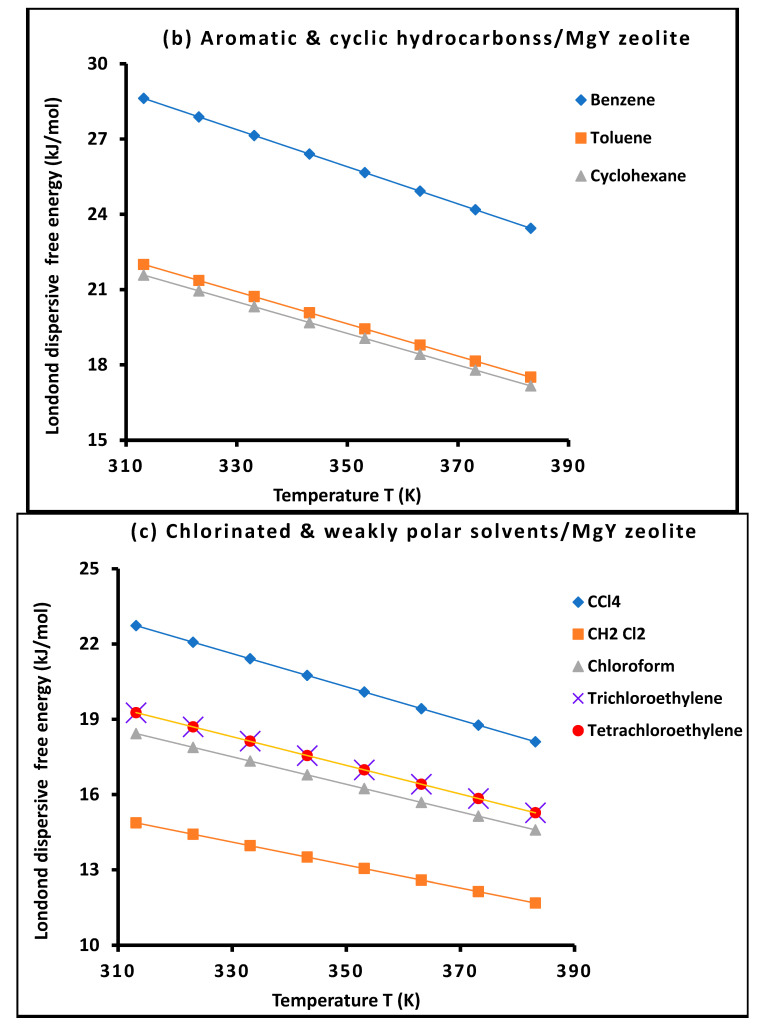

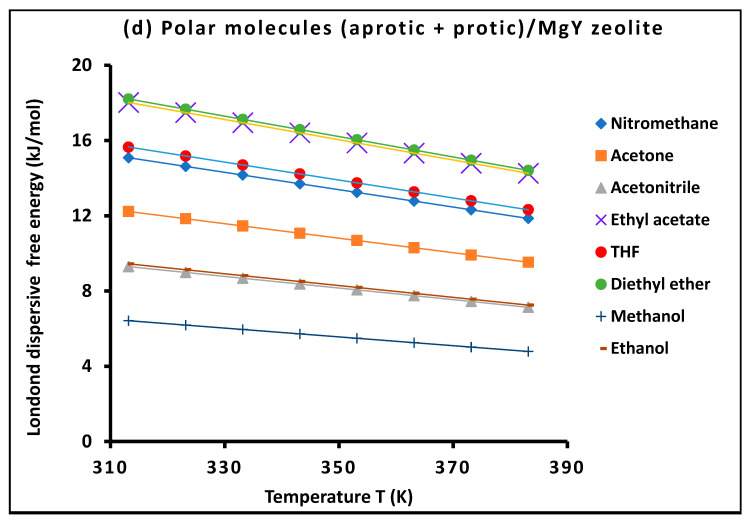
Temperature dependence of the London dispersive free energy of adsorption $\Delta G_{a}^{d}\left(T\right)$ for probe molecules on MgY zeolite, grouped according to physicochemical families: (**a**) n-alkanes, (**b**) aromatic and cyclic hydrocarbons, (**c**) chlorinated compounds, (**d**) polar solvents (aprotic and protic). This classification highlights the influence of molecular polarity and interaction type on adsorption behavior.

**Figure 2:**
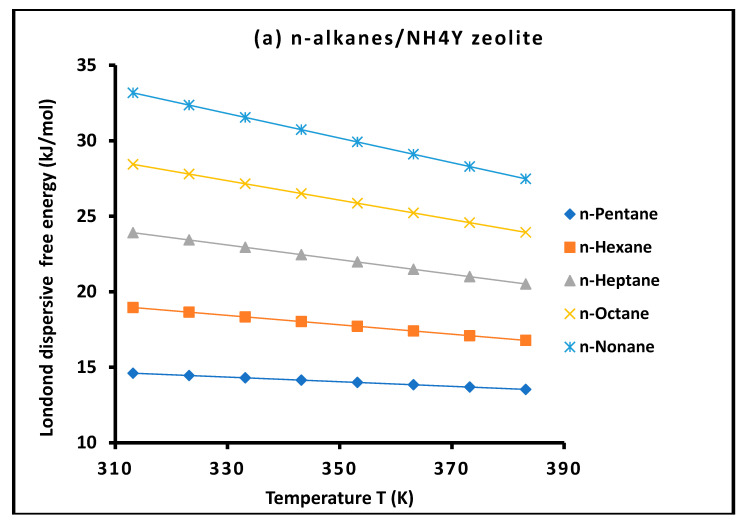

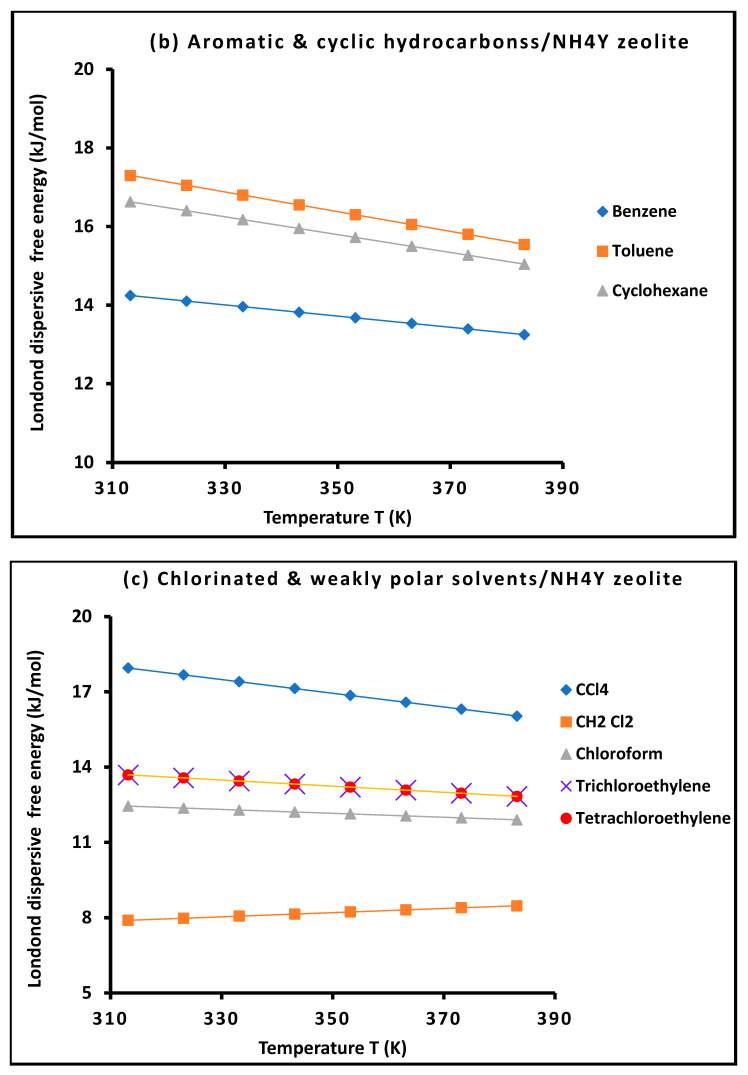

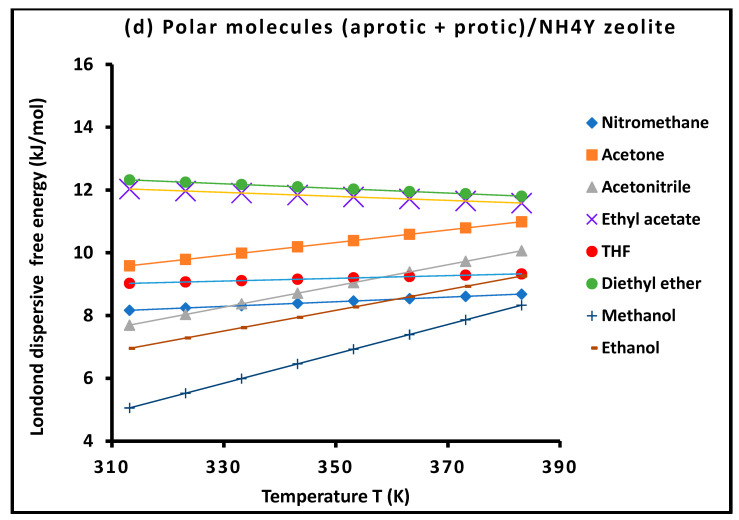
Temperature dependence of the London dispersive free energy of adsorption $\Delta G_{a}^{d}\left(T\right)$ for probe molecules on NH_4_Y zeolite, organized by physicochemical families. (**a**) n-alkanes, (**b**) aromatic and cyclic hydrocarbons, (**c**) chlorinated compounds, (**d**) polar solvents (aprotic and protic). The grouping emphasizes the contrast between classical dispersive behavior and non-classical trends observed for polar molecules, including positive temperature dependence for certain probes.

For all investigated solvent–solid systems, the variation in the dispersive free energy $\Delta G_{a}^{d}(T)$ with temperature, as presented in Figure 1 and Figure 2, exhibits excellent linear behavior over the entire temperature range studied. The corresponding coefficients of determination are consistently very high, typically exceeding $R^{2}>0.9997$, reflecting the remarkable precision of the experimental measurements.

This pronounced linearity validates the thermodynamic treatment adopted in this work and confirms that adsorption occurs within the **Henry regime**, where the surface coverage remains sufficiently low to neglect lateral interactions between adsorbed molecules. Under these conditions, the measured free energy corresponds exclusively to probe–surface interactions, ensuring the reliability of the derived thermodynamic parameters.

Moreover, the observed linear dependence further supports the assumption that dispersive interactions can be consistently described using a temperature-dependent formulation. This behavior highlights the internal consistency of the present methodology and reinforces its robustness for the quantitative characterization of surface energetics.

For clarity and to highlight the role of molecular properties, the probe molecules in Figure 1 and Figure 2 were grouped according to their physicochemical characteristics, including nonpolar n-alkanes, aromatic/cyclic hydrocarbons, chlorinated compounds, and polar solvents. This classification allows a clearer visualization of adsorption trends and facilitates the identification of distinct thermodynamic behaviors associated with different interaction mechanisms.

**Figure 3:**
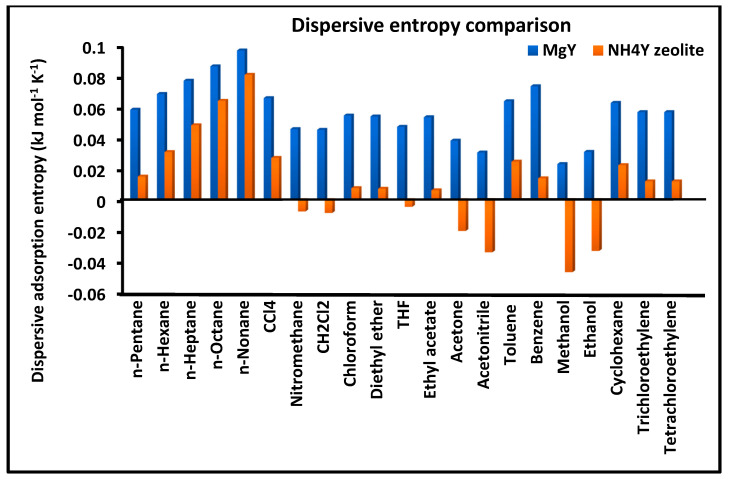
Comparison between the dispersive entropy of adsorption of solvents on MgY and NH_4_Y zeolites.

Figure 3 presents a comparative analysis of the dispersive entropy of adsorption $\Delta S_{a}^{d}$ for the investigated solvents on MgY and NH_4_Y zeolite surfaces. The entropy values are derived from the temperature dependence of the dispersive free energy and provide insight into the degree of molecular ordering and configurational freedom during adsorption.

The results reveal a clear and systematic difference between the two zeolites. For most solvents, MgY exhibits higher dispersive entropy values compared to NH_4_Y, indicating a more pronounced thermally activated response of the adsorption process. This behavior suggests that adsorption on MgY involves a more structured interaction field, where thermal fluctuations significantly influence intermolecular interactions. In contrast, the lower and more variable entropy values observed for NH_4_Y reflect a more complex adsorption environment, in which polar interactions and hydrogen bonding constrain molecular motion and reduce configurational entropy. In some cases, the presence of low or even negative entropy contributions further supports the existence of restricted adsorption geometries and strong localized interactions on NH_4_Y.

**Figure 4:**
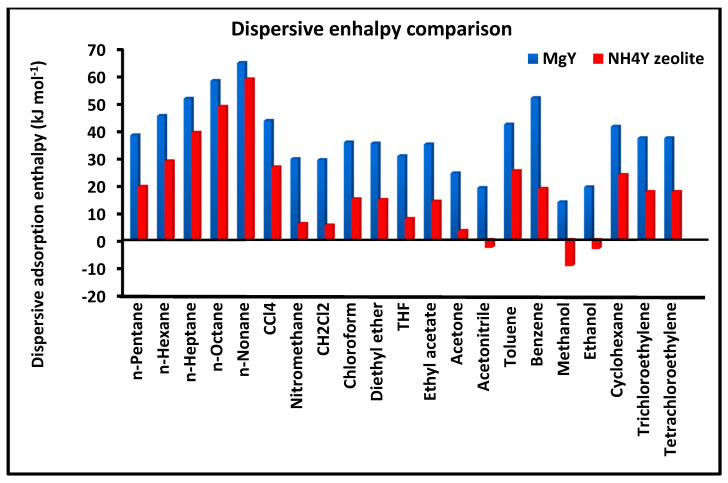
Comparison between the London dispersive energy of adsorption of solvents on MgY and NH_4_Y zeolites.

Figure 4 compares the London dispersive free energy of adsorption $\Delta G_{a}^{d}$ for the different probe molecules on MgY and NH_4_Y surfaces. This parameter reflects the strength of dispersive interactions and constitutes a key descriptor of surface energetics.

The comparison highlights a consistent trend in which the magnitude of $\Delta G_{a}^{d}$ is systematically higher on MgY than on NH_4_Y for all investigated solvents. This observation confirms that MgY possesses a stronger dispersive interaction field, which can be attributed to enhanced polarization effects induced by Mg^2+^ cations. The higher dispersive free energy indicates a stronger stabilization of adsorbed molecules and a more energetically structured surface. In contrast, the lower values observed for NH_4_Y reflect weaker dispersive interactions, consistent with a surface where polar and hydrogen-bonding interactions play a more dominant role. The clear separation between the two sets of data demonstrates the ability of the present methodology to discriminate between surfaces based on their dispersive interaction strength.

**Figure 5:**
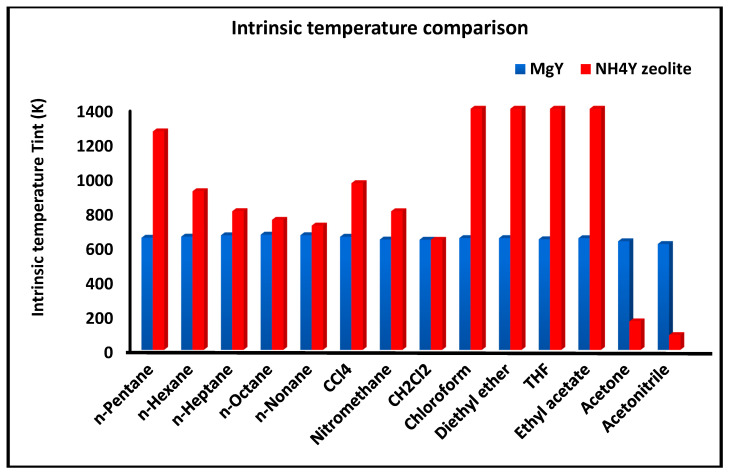
Comparison between the dispersive intrinsic temperature of solvents on MgY and NH_4_Y zeolites.

Figure 5 presents the intrinsic (or compensation) temperature $T_{\mathrm{int}}$ associated with the dispersive component of adsorption for the investigated solvents on MgY and NH_4_Y. This parameter, defined as the ratio $\Delta H_{a}^{d}/\Delta S_{a}^{d}$, reflects the balance between enthalpic and entropic contributions to adsorption.

The comparison reveals that MgY generally exhibits lower and more narrowly distributed $T_{\mathrm{int}}$ values, indicating a more uniform thermodynamic behavior across the different probes. This suggests that adsorption on MgY is governed by a consistent balance between enthalpic stabilization and entropic contributions, characteristic of a homogeneous interaction field. In contrast, NH_4_Y displays higher and more widely dispersed intrinsic temperatures, reflecting a greater variability in the thermodynamic balance. This behavior is indicative of a more heterogeneous surface, where adsorption mechanisms vary significantly depending on the nature of the probe molecule. The broader distribution of $T_{\mathrm{int}}$ values on NH_4_Y further supports the presence of multiple interaction regimes, including strong polar interactions and hydrogen bonding, which introduce additional complexity into the adsorption process.

Overall, the combined analysis of Figure 3, Figure 4 and Figure 5 provides a coherent and complementary picture of the dispersive thermodynamics, clearly demonstrating the more structured and energetically uniform nature of MgY compared to the more flexible, heterogeneous, and interaction-sensitive behavior of NH_4_Y.

### 4.3. Thermodynamic Analysis of London Dispersive Adsorption on MgY and NH_4_Y Zeolites

The thermodynamic parameters reported in Table S4 provide a comprehensive and quantitative description of the London dispersive interactions governing the adsorption of both nonpolar (n-alkanes) and polar solvents on MgY and NH_4_Y zeolites. The excellent linearity observed for all systems (R^2^ ≈ 1) confirms that adsorption occurs in the Henry regime and validates the robustness of the present thermodynamic framework over the investigated temperature range. Such behavior is consistent with classical IGC observations reported in the literature for well-defined adsorption systems [25,26,27,28,29], while extending them through a more rigorous thermodynamic interpretation.

#### 4.3.1. Dispersive Interaction Strength: Comparison Between Nonpolar and Polar Solvents

A first important result concerns the comparison between solvent families. For both zeolites, the homologous series of n-alkanes exhibits a clear and systematic increase in dispersive adsorption enthalpy $\Delta H_{a}^{d}$ with increasing carbon number, reflecting the progressive enhancement of London interactions with molecular size and polarizability. This trend is well established in the literature [27,30,31,32] and is fully confirmed by the present results.

For MgY, $\Delta H_{a}^{d}$ increases from 38.2 kJ·mol^−1^ (n-pentane) to 64.6 kJ·mol^−1^ (n-nonane), while for NH_4_Y the corresponding values range from 19.4 to 58.6 kJ·mol^−1^. The nearly constant increment between successive n-alkanes indicates a regular and homogeneous interaction field, particularly for MgY, suggesting adsorption on energetically similar sites.

In contrast, polar solvents exhibit significantly lower dispersive enthalpy values than n-alkanes of comparable size. This behavior reflects the fact that their total interaction energy is partitioned between dispersive and specific (acid–base) contributions, as widely reported in IGC studies [25,26,27,28,29]. The present methodology allows this separation explicitly, revealing that the dispersive contribution alone is reduced for polar molecules due to the increasing role of specific interactions.

#### 4.3.2. Fundamental Difference Between MgY and NH_4_Y Surfaces

A striking result emerging from Table S4 is the systematic difference between MgY and NH_4_Y for all investigated probes. For a given molecule, the dispersive enthalpy is consistently higher on MgY than on NH_4_Y, demonstrating a stronger dispersive interaction field.

This behavior is consistent with the electronic structure of the two materials. MgY, containing Mg^2+^ cations, generates strong local electrostatic fields that enhance polarization and induction effects, thereby reinforcing London dispersion interactions. Similar effects have been reported for cation-exchanged zeolites and metal oxides, where increased charge density leads to enhanced polarizability of the adsorbate–surface system [22,23,24].

In contrast, NH_4_Y exhibits a softer and more deformable electronic environment, where dispersive interactions are present but not amplified by strong electrostatic contributions. This leads to lower dispersive energies and a more flexible adsorption environment.

#### 4.3.3. Behavior of Polar Molecules and Deviation from Classical Trends

The behavior of polar probes further highlights the complexity of adsorption mechanisms. On MgY, polar molecules such as acetone, acetonitrile, and alcohols exhibit lower dispersive enthalpies compared to n-alkanes, consistent with the redistribution of interaction energy toward specific acid–base contributions.

In contrast, adsorption on NH_4_Y exhibits atypical thermodynamic behavior for several polar probes, including very low dispersive enthalpies, reduced or even negative entropic contributions, and, in some cases, a positive temperature dependence of $\Delta G_{a}^{d}(T)$.

Such deviations from classical behavior have been reported for strongly interacting systems involving hydrogen bonding or confinement effects [12,13,14]. In the present case, they reflect a strong competition between dispersive and specific interactions, as well as possible molecular reorganization at the interface.

These results clearly demonstrate that dispersive interactions cannot be interpreted independently for polar systems and highlight the necessity of a complete thermodynamic separation, as achieved by the present methodology.

#### 4.3.4. Enthalpy–Entropy Compensation and Intrinsic Temperature

A key feature of the results is the existence of a well-defined enthalpy–entropy compensation effect for dispersive adsorption. For MgY, the intrinsic temperature $T_{\mathrm{int}}$ values are narrowly distributed (≈640–670 K), indicating a highly coherent thermodynamic behavior and a uniform interaction mechanism.

Such compensation behavior has been widely reported in adsorption systems and is generally associated with a common underlying interaction mechanism [25,26,27,28,29]. In the present case, it reflects the structured nature of the MgY surface, where variations in enthalpy are systematically balanced by entropy changes.

In contrast, NH_4_Y exhibits significantly higher and more dispersed $T_{\mathrm{int}}$ values, often exceeding 1000 K or becoming poorly defined. This dispersion indicates a heterogeneous adsorption landscape and a more complex balance between energetic and configurational contributions.

From a physical standpoint, this difference indicates that MgY behaves as a more energetically structured surface with well-defined interaction sites, whereas NH_4_Y exhibits a more flexible and heterogeneous adsorption environment.

#### 4.3.5. Implications for Adsorption Mechanisms

The combined analysis of enthalpy, entropy, and compensation temperature provides deep insight into the adsorption mechanisms. On MgY, adsorption is governed by strong, well-defined interaction sites, leading to high enthalpic stabilization and relatively ordered adsorption states.

In contrast, adsorption on NH_4_Y is weaker and more sensitive to molecular structure and environmental fluctuations, resulting in lower enthalpic contributions and more variable, and sometimes constrained, entropy effects. This behavior is consistent with adsorption dominated by hydrogen bonding and protonic interactions, as reported for ammonium-containing zeolites [6,13,15].

This distinction highlights two fundamentally different adsorption regimes: a structured, electrostatically enhanced regime (MgY) and a more flexible, interaction-sensitive regime (NH_4_Y).

#### 4.3.6. Dispersive Adsorption Compensation

**Figure 6:**
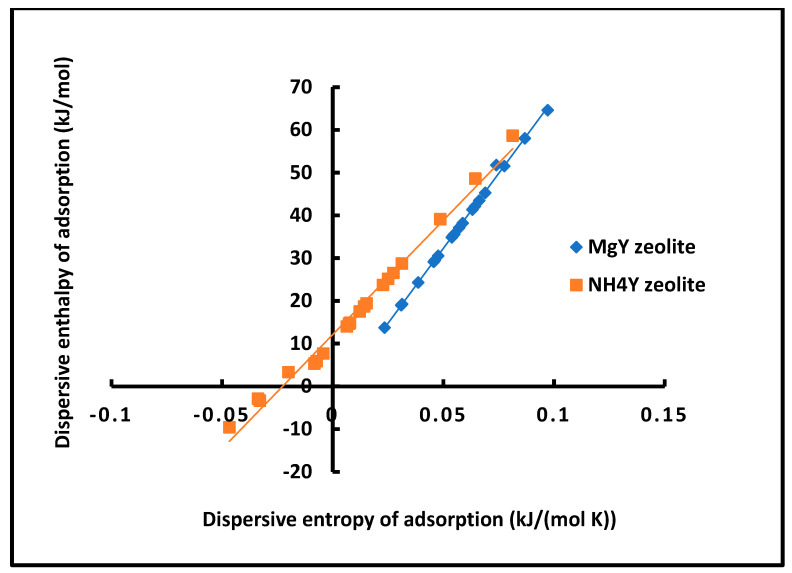
Enthalpy–entropy compensation for dispersive adsorption. Linear relationships between dispersive adsorption enthalpy, $-\Delta H_{a}^{d}$, and entropy, $-\Delta S_{a}^{d}$, for probe molecules adsorbed on MgY and NH_4_Y. The slopes of the regression lines correspond to the dispersive isokinetic temperature, evidencing a well-defined thermodynamic compensation effect.

The enthalpy–entropy compensation relationships for dispersive adsorption are presented in Figure 6 and summarized in Table 1. The strong linear correlations observed for both materials confirm the existence of a well-defined thermodynamic compensation effect.

The corresponding isokinetic temperatures are:

- $T_{\mathrm{iso}}$ = 701.4 K for MgY;
- $T_{\mathrm{iso}}$ = 534.6 K for NH_4_Y.

The higher value for MgY reflects a stronger and more structured interaction field, whereas the lower value for NH_4_Y indicates a more flexible adsorption environment.

The excellent linearity (R^2^ = 0.9975 for MgY and 0.9876 for NH_4_Y) confirms the reliability of the thermodynamic analysis. The slightly lower correlation for NH_4_Y further supports the presence of surface heterogeneity.

From a broader perspective, these results demonstrate that even dispersive interactions exhibit a coherent thermodynamic structure governed by electronic properties, in agreement with advanced theoretical approaches based on London and Hamaker formalisms [33,34,35].

In contrast, the lower value of $T_{iso}$ for NH_4_Y indicates a more flexible and less constrained adsorption environment, where dispersive interactions are weaker and the configurational freedom of adsorbed molecules plays a more significant role. This behavior is consistent with the softer electronic structure associated with ammonium-containing surfaces.

The excellent linear correlations obtained for both systems, with coefficients of determination $R^{2}=0.9975\;$ for MgY and $R^{2}=0.9876\;$ for NH_4_Y, further confirm the robustness of the compensation effect and the reliability of the extracted thermodynamic parameters. The slightly lower correlation coefficient observed for NH_4_Y reflects a higher degree of heterogeneity and variability in adsorption sites, which may induce deviations from ideal compensation behavior.

From a broader perspective, the existence of such well-defined compensation relationships for the dispersive component alone constitutes a significant result. It demonstrates that London dispersion interactions, often considered purely additive and non-specific, exhibit a coherent thermodynamic structure governed by the electronic properties of both the surface and the adsorbate.

This finding provides strong support for the present methodology, which treats dispersive interactions within a unified thermodynamic framework and reveals their intrinsic coupling with entropy. It also highlights the fundamental difference between MgY and NH_4_Y zeolites, not only in terms of interaction strength but also in the nature and organization of their adsorption energy landscapes.

### 4.4. Thermodynamic Parameters of Polar Adsorption

#### 4.4.1. Comparison Between Polar Energy Components

**Figure 7:**
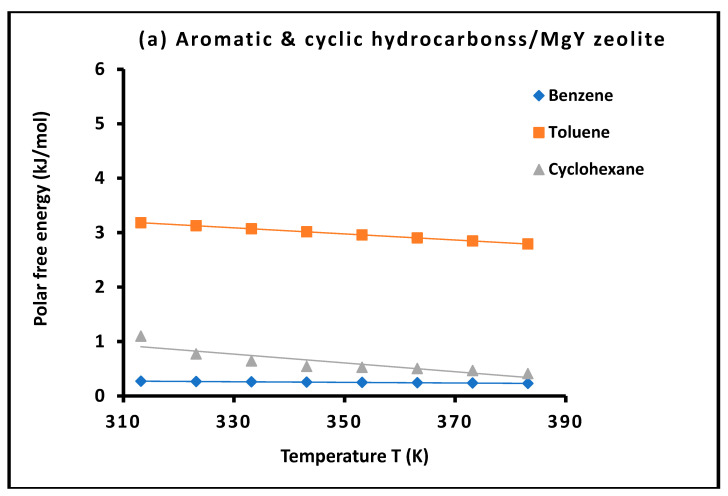

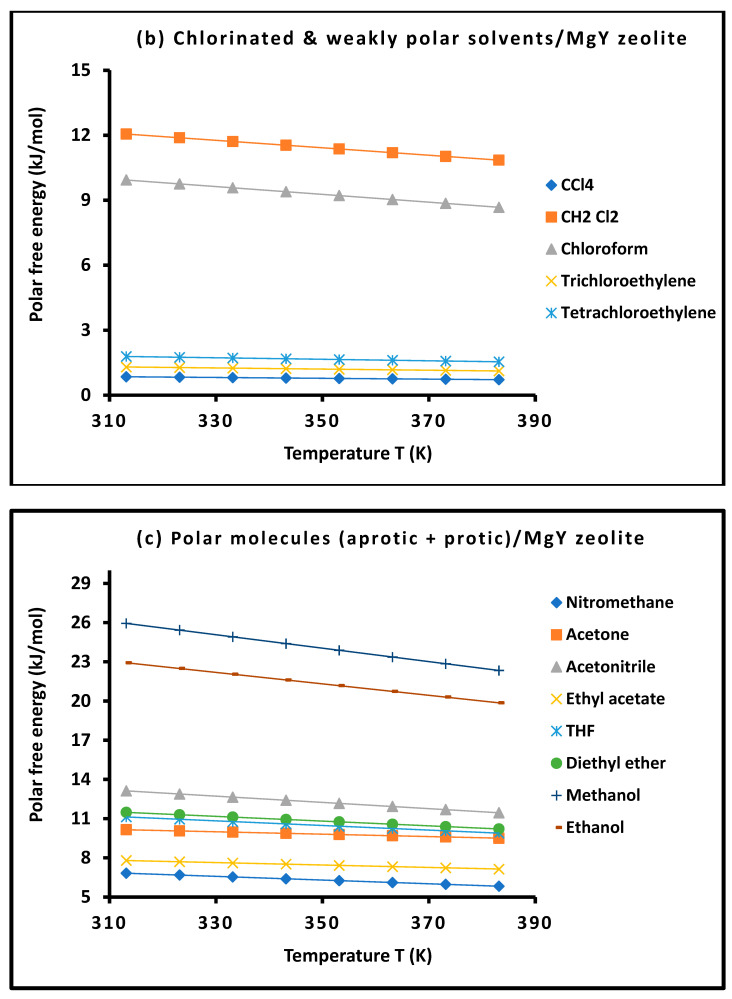
Temperature dependence of the polar free energy of adsorption, $\Delta G_{a}^{p}(T)$, for probe molecules adsorbed on MgY zeolite. For clarity, the data are grouped according to physicochemical families: (**a**) aromatic and cyclic hydrocarbons, (**b**) chlorinated compounds, and (**c**) polar aprotic and protic solvents. This representation highlights the progressive decrease in polar interactions with temperature and reveals the strong contribution of donor–acceptor and hydrogen-bonding interactions for highly polar probes, compared with the weak polar contribution of nonpolar or weakly polar molecules.

**Figure 8:**
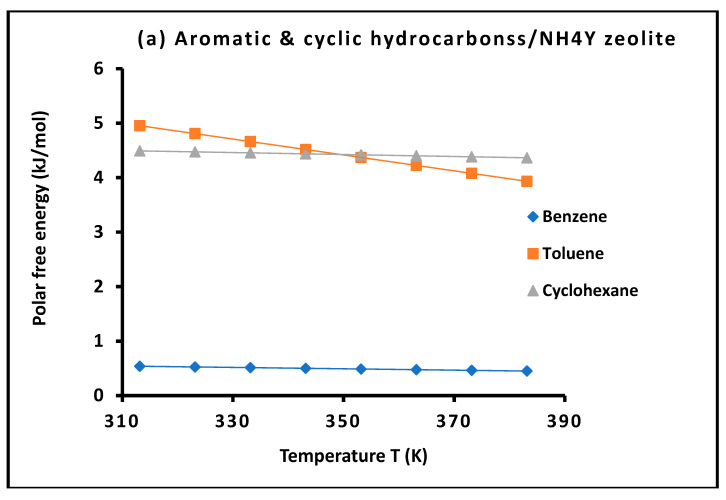

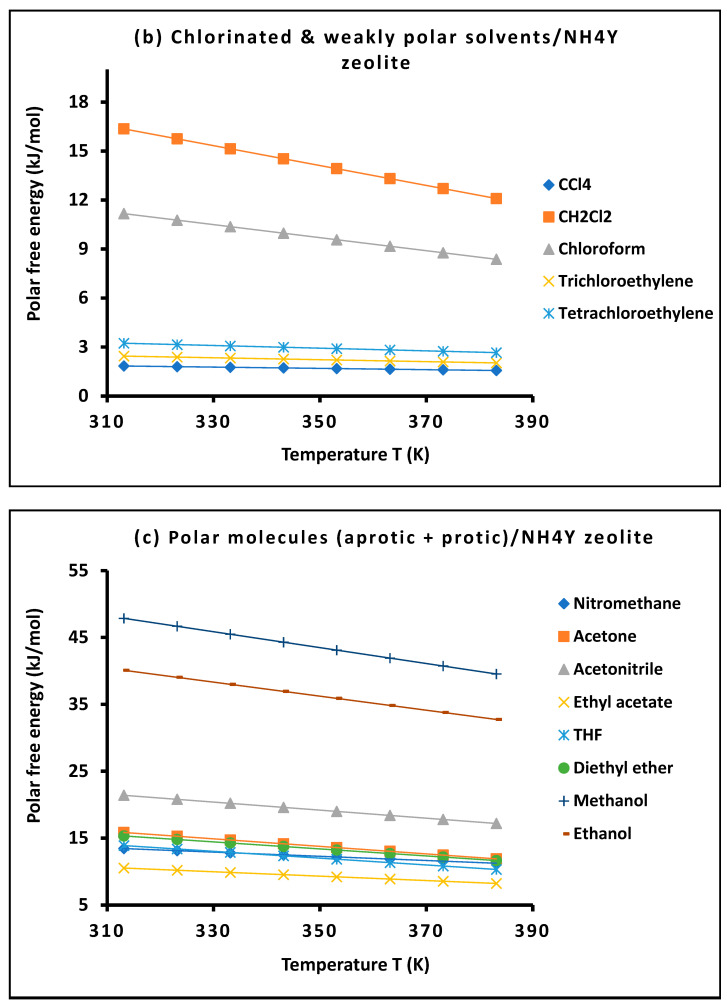
Temperature dependence of the polar free energy of adsorption, $\Delta G_{a}^{p}(T)$, for probe molecules adsorbed on NH_4_Y zeolite, organized according to physicochemical families as in Figure 7. (**a**) aromatic and cyclic hydrocarbons, (**b**) chlorinated compounds, and (**c**) polar aprotic and protic solvents. The grouped representation emphasizes the stronger and more heterogeneous polar interactions compared with MgY, particularly for hydrogen-bonding and highly polar molecules. The variability of slopes and magnitudes reflects the complex interplay between protonic sites, hydrogen bonding, and dipolar interactions governing adsorption on NH_4_Y.

The thermodynamic parameters associated with the polar component of adsorption on MgY and NH_4_Y zeolites, reported in Table S5, provide detailed insight into the specific interactions governing probe–surface affinity beyond London dispersion. The excellent linearity of the temperature dependence of the polar free energy $\Delta G_{a}^{p}(T)$, illustrated in Figure 7 and Figure 8 with coefficients of determination systematically equal or very close to unity, confirms both the high precision of the measurements and the validity of the thermodynamic decomposition adopted in this work.

To improve the readability of the polar adsorption results and to highlight the role of molecular structure, the variations in the polar free energy of adsorption, $\Delta G_{a}^{p}(T)$, were reorganized into two separate figures for MgY and NH_4_Y zeolites. As for the dispersive contribution, the probe molecules were grouped according to their physicochemical nature, excluding the n-alkanes because their polar contribution is considered negligible. This classification allows a clearer comparison between chlorinated compounds, aromatic and cyclic molecules, polar aprotic solvents, and hydrogen-bonding probes.

Figure 7 presents the temperature dependence of the polar free energy of adsorption, $\Delta G_{a}^{p}(T)$, for probe molecules adsorbed on MgY zeolite. The curves show a systematic decrease of $\Delta G_{a}^{p}(T)$ with increasing temperature for almost all polar probes, confirming that specific interactions become progressively weaker as thermal motion increases.

A clear hierarchy of interaction strength is observed. Alcohols, particularly methanol and ethanol, exhibit the highest polar free energies, indicating strong specific interactions with MgY surface sites. This behavior reflects the ability of alcohol molecules to interact through hydrogen bonding and donor–acceptor interactions with Lewis acidic Mg^2+^ centers and framework oxygen atoms. Strong polar contributions are also observed for acetonitrile, dichloromethane, THF, diethyl ether, acetone, and chloroform, confirming the importance of electron donor interactions and dipolar effects.

In contrast, benzene, cyclohexane, trichloroethylene, tetrachloroethylene, and CCl_4_ exhibit much lower polar contributions, as expected from their weak specific interaction ability. Their behavior confirms that their adsorption is mainly governed by dispersive interactions, with only minor polar or specific contributions.

Overall, Figure 7 shows that MgY provides a structured Lewis-type adsorption environment, where the polar free energy is strongly controlled by the donor ability, dipolar character, and hydrogen-bonding capacity of the probe molecules.

Figure 8 shows the temperature dependence of $\Delta G_{a}^{p}(T)$ for the same polar probes adsorbed on NH_4_Y zeolite. Compared with MgY, NH_4_Y exhibits stronger and more heterogeneous polar interactions for several solvents, reflecting the important role of ammonium species, protonic sites, and hydrogen-bonding effects.

The largest polar free energies are observed for strongly interacting molecules such as methanol, ethanol, acetone, ethyl acetate, acetonitrile, chloroform, and diethyl ether. These results indicate that NH_4_Y possesses a highly polar adsorption environment, where specific interactions are strongly influenced by hydrogen bonding, proton-donor character, and cooperative effects within the zeolite pores.

The decrease of $\Delta G_{a}^{p}(T)$ with temperature confirms that polar interactions are enthalpically favored but progressively weakened by thermal agitation. However, compared with MgY, the amplitudes and slopes of the curves are more variable, indicating that adsorption on NH_4_Y is governed by a broader distribution of interaction sites and by a more complex balance between polar, hydrogen-bonding, and confinement effects.

Weakly polar molecules such as toluene, benzene, cyclohexane, and trichloroethylene display smaller polar contributions, confirming that their adsorption remains largely dispersive. Nevertheless, their non-zero polar energies indicate that even weakly polarizable molecules can interact specifically with the ammonium-modified zeolite surface.

Taken together, Figure 7 and Figure 8 provide a clear comparative picture of the polar adsorption behavior of MgY and NH_4_Y zeolites. MgY exhibits a more structured and regular polar interaction field, mainly governed by localized Lewis acidity and electrostatic polarization, whereas NH_4_Y displays stronger, more variable, and more complex polar interactions associated with hydrogen bonding, protonic effects, and surface heterogeneity. This comparison confirms that the polar contribution cannot be treated as a simple residual term but represents a key thermodynamic descriptor of surface chemistry. The grouped graphical presentation therefore strengthens the interpretation of the adsorption mechanisms and clearly demonstrates the ability of the present methodology to distinguish between dispersive, polar, and specific interaction regimes.

**Figure 9:**
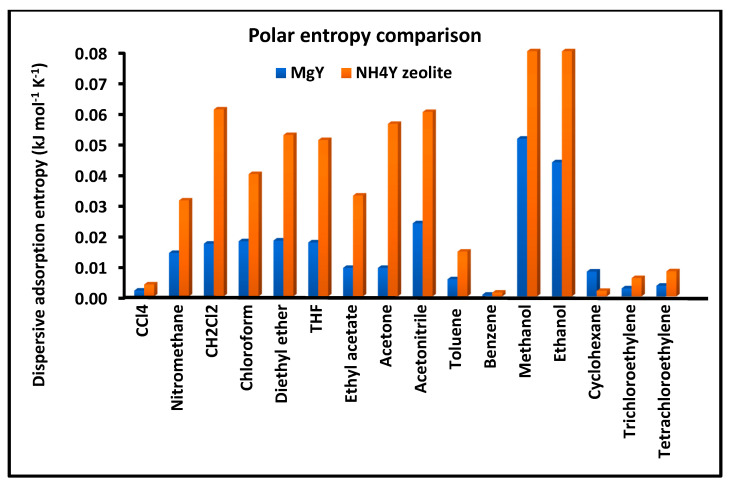
Comparison between the polar entropy of adsorption of solvents on MgY and NH_4_Y zeolites.

Figure 9 presents a comparative analysis of the polar entropy of adsorption $\Delta S_{a}^{p}$ for the investigated solvents on MgY and NH_4_Y zeolite surfaces. This parameter reflects the degree of configurational freedom and molecular organization associated with specific (acid–base) interactions during adsorption.

The comparison reveals a pronounced difference between the two materials. NH_4_Y generally exhibits higher absolute values of polar entropy, often accompanied by significant variability among the probes. This behavior reflects a complex adsorption environment in which hydrogen bonding and proton-donor interactions introduce strong constraints on molecular mobility, leading in some cases to reduced or even negative entropic contributions. In contrast, MgY displays more moderate and uniformly distributed entropy values, indicating a more regular adsorption behavior governed by localized Lewis-type interactions. The narrower distribution observed for MgY suggests a more homogeneous interaction field, whereas the broader variability on NH_4_Y highlights the strong sensitivity of polar adsorption to molecular structure and interaction specificity.

**Figure 10:**
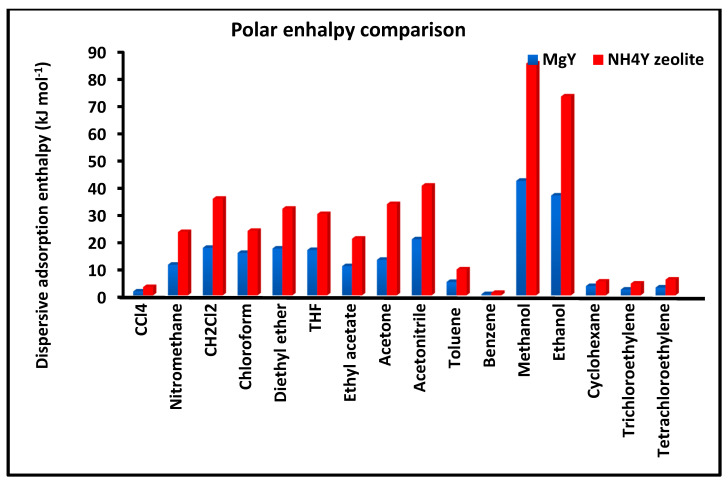
Comparison between the polar enthalpy of adsorption of solvents on MgY and NH_4_Y zeolites.

Figure 10 compares the polar enthalpy of adsorption $\Delta H_{a}^{p}\;$ for the investigated solvents on MgY and NH_4_Y zeolites. This parameter quantifies the strength of specific interactions, including donor–acceptor interactions, hydrogen bonding, and electrostatic contributions.

A clear distinction is observed between the two zeolites. NH_4_Y exhibits significantly higher polar enthalpy values for most solvents, indicating stronger specific interactions. This behavior is consistent with the presence of protonated ammonium species, which promote hydrogen bonding and enhance the interaction strength with polar probes. In contrast, MgY generally shows lower polar enthalpy values, reflecting adsorption dominated by Lewis acid–base interactions with less pronounced hydrogen-bonding contributions. The results therefore confirm that NH_4_Y provides a more strongly interacting polar surface, while MgY exhibits a more moderate and structurally defined interaction field. This distinction is in full agreement with the trends observed in the Lewis acid–base parameters.

**Figure 11:**
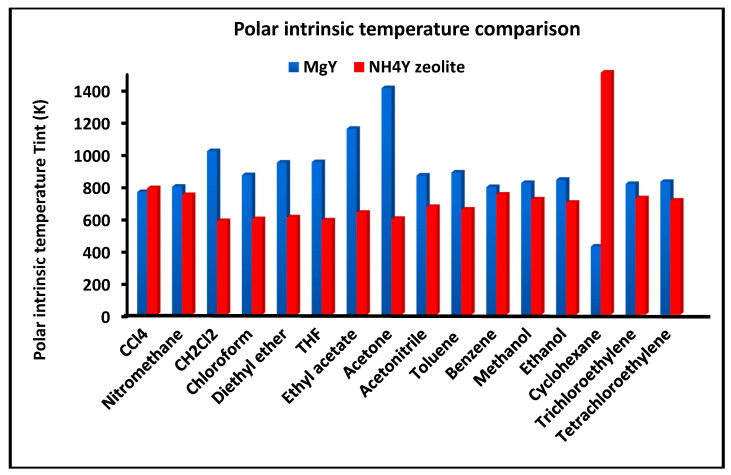
Comparison between the polar intrinsic temperature of solvents on MgY and NH_4_Y zeolites.

Figure 11 presents the intrinsic (or compensation) temperature $T_{\mathrm{int}}$ associated with the polar component of adsorption for the investigated solvents on MgY and NH_4_Y. This parameter reflects the balance between enthalpic stabilization and entropic contributions in polar adsorption processes.

The comparison highlights a fundamental difference in thermodynamic behavior. MgY exhibits relatively consistent intrinsic temperature values, indicating a stable balance between enthalpic and entropic contributions across the different probes. This behavior is characteristic of a structured and energetically coherent adsorption environment. In contrast, NH_4_Y displays a broader distribution of $T_{\mathrm{int}}$ values, reflecting significant variability in the relative contributions of enthalpy and entropy. In some cases, the absence of a well-defined intrinsic temperature further indicates deviations from classical compensation behavior, consistent with strong nonlinear effects and heterogeneous interaction mechanisms. This variability confirms that polar adsorption on NH_4_Y is governed by a more complex interplay of hydrogen bonding, proton transfer effects, and cooperative interactions.

Taken together, Figure 9, Figure 10 and Figure 11 provide a coherent and detailed picture of polar adsorption thermodynamics, demonstrating that NH_4_Y exhibits stronger, more variable, and highly nonlinear polar interactions, whereas MgY displays a more structured and thermodynamically uniform behavior dominated by classical Lewis acid–base interactions.

#### 4.4.2. Magnitude and Nature of Polar Interactions

A first important observation concerns the markedly different magnitude of the polar adsorption enthalpies $\Delta H_{a}^{p}$ for the two zeolites. For MgY, the polar enthalpies span a relatively moderate range, typically between ~0.5 and 42 kJ·mol^−1^, with the highest values observed for strongly interacting probes such as methanol (42.1 kJ·mol^−1^) and ethanol (36.6 kJ·mol^−1^). These values reflect the presence of well-defined Lewis acid sites associated with Mg^2+^ cations, which interact preferentially with electron donor molecules.

In contrast, NH_4_Y exhibits significantly higher polar adsorption enthalpies for many probes, reaching 85.1 kJ·mol^−1^ for methanol and 73.0 kJ·mol^−1^ for ethanol, as well as elevated values for acetonitrile and dichloromethane. This behavior clearly indicates that NH_4_Y possesses stronger specific interactions, which can be attributed to the presence of protonic (Brønsted-type) sites and enhanced hydrogen-bonding capability.

This result highlights a fundamental distinction between the two systems: MgY is governed primarily by localized Lewis acidic sites and strong electrostatic polarization effects, while NH_4_Y displays interaction mechanisms dominated by hydrogen bonding and proton-donor character, resulting in enhanced affinity and higher adsorption energies for polar molecules.

#### 4.4.3. Entropic Contributions and Molecular Organization

The entropy changes associated with polar adsorption also reveal important differences in the adsorption mechanisms. On MgY, the entropy values remain relatively moderate and positive, indicating that adsorption is accompanied by a controlled loss of degrees of freedom, consistent with localized interactions at specific active sites.

On NH_4_Y, however, the entropy variations are significantly larger in magnitude, particularly for strongly interacting probes such as alcohols. This suggests that adsorption involves more pronounced structural rearrangements and configurational constraints, likely associated with hydrogen-bond networks and cooperative interactions within the zeolite pores.

Interestingly, several probes exhibit atypical thermodynamic behavior, including very small or even sign-changing temperature coefficients in the case of NH_4_Y. These deviations reflect the complexity of the adsorption process, where competing interactions (dispersion, acid–base, and hydrogen bonding) coexist and may partially compensate each other.

#### 4.4.4. Temperature Dependence and Stability of Polar Interactions

The linear dependence of $\Delta G_{a}^{p}(T)$ on temperature for all probes confirms that the polar interactions can be reliably described within a classical thermodynamic framework. However, the slopes of these relationships, which correspond to entropy contributions, differ significantly between MgY and NH_4_Y.

For MgY, the relatively uniform slopes indicate that polar interactions are governed by a consistent mechanism across different probes. In contrast, NH_4_Y shows a broader dispersion of slopes, reflecting a greater sensitivity of adsorption to temperature and a more heterogeneous interaction landscape.

This difference further supports the view that MgY provides a structured and energetically homogeneous surface, characterized by more uniform interaction sites, whereas NH_4_Y exhibits a more flexible and dynamically responsive adsorption environment, reflecting greater sensitivity to molecular polarity, hydrogen bonding, and temperature-induced reorganization.

#### 4.4.5. Enthalpy–Entropy Compensation for Polar Adsorption

**Figure 12:**
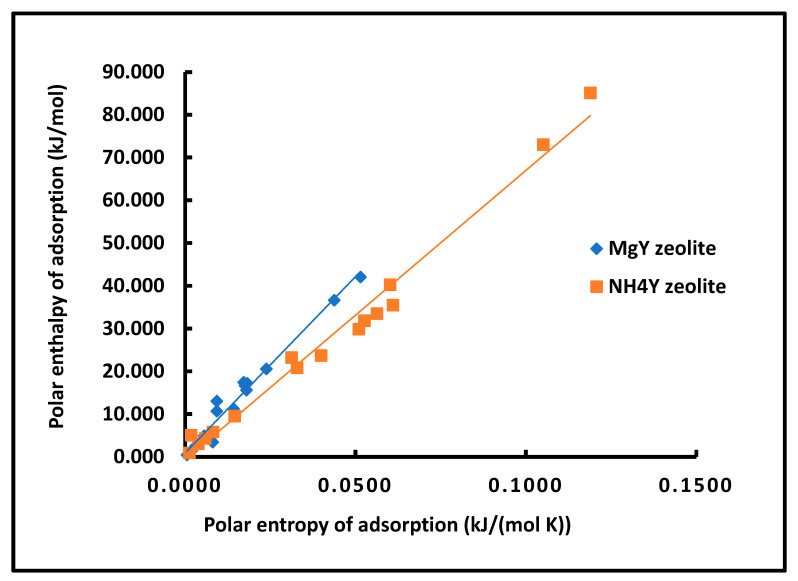
Enthalpy–entropy compensation for polar adsorption. Correlation between polar adsorption enthalpy, $-\Delta H_{a}^{p}$, and entropy, $-\Delta S_{a}^{p}$, for the investigated probe molecules on MgY and NH_4_Y. The linear behavior indicates the existence of a thermodynamic compensation effect associated with specific (acid–base and hydrogen-bonding) interactions.

The enthalpy–entropy compensation plots presented in Figure 12 reveal well-defined linear relationships between $\left(-\Delta H_{a}^{p}\right)$ and $\left(-\Delta S_{a}^{p}\right)$ for both zeolites, confirming the existence of a compensation effect for the polar component of adsorption.

The corresponding regression equations, summarized in Table 2, lead to the determination of the isokinetic temperature $T_{\mathrm{iso}}$, which reflects the intrinsic thermodynamic balance of the system:

- $T_{\mathrm{iso}}=825.2\;K$ for MgY;
- $T_{\mathrm{iso}}=676.9\;K$ for NH_4_Y.

**Table 2:** Enthalpy–entropy compensation parameters for polar adsorption. Linear relationships between polar adsorption enthalpy $\left(-\Delta H_{a}^{p}\right)$ and entropy $\left(-\Delta S_{a}^{p}\right)$ for MgY and NH_4_Y zeolites, including the corresponding isokinetic temperature $T_{\mathrm{iso}}$ and regression coefficients $R^{2}$.

Materials	Polar Adsorption	$T_{iso}$	R^2^
MgY	$\left(-\Delta H^{d}\right)$$\;=\;825.2\;(-\Delta S^{d})$ − 0.886	825.2	0.9735
NH_4_Y	$\left(-\Delta H^{d}\right)$$\;=\;676.9\;(-\Delta S^{d})$ − 0.701	676.9	0.9840

The higher compensation temperature observed for MgY indicates a stronger coupling between enthalpic and entropic contributions, consistent with the presence of well-defined and energetically constrained adsorption sites. Conversely, the lower $T_{\mathrm{iso}}$ for NH_4_Y reflects a more flexible adsorption environment, where entropy plays a more prominent role.

The excellent correlation coefficients ($R^{2}=0.9735$ for MgY and $R^{2}=0.9840$ for NH_4_Y) confirm the robustness of this compensation behavior, despite the complexity of the underlying interactions.

#### 4.4.6. Comparison with Dispersive Contributions

A comparison with the dispersive thermodynamic parameters (Section 4.2) reveals a fundamental difference in the nature of adsorption. Dispersive interactions exhibit a highly structured and uniform behavior, particularly on MgY, reflecting a well-defined interaction field, whereas polar interactions display greater variability and pronounced sensitivity to molecular properties, especially on NH_4_Y, indicating a more complex and heterogeneous adsorption environment.

This contrast demonstrates that while London dispersion is primarily governed by **electronic polarizability**, polar adsorption depends on **specific chemical interactions**, including Lewis acid–base effects and hydrogen bonding.

#### 4.4.7. Methodological Significance and Implications

The results presented here clearly demonstrate the strength and versatility of the present IGC-based methodology, which enables the quantitative separation of dispersive and polar (specific) contributions, provides independent access to the enthalpic and entropic components of adsorption, and reveals subtle yet significant differences in surface reactivity that cannot be captured by conventional approaches. By establishing a direct relationship between adsorption thermodynamics and surface electronic properties, the method offers a deeper and more physically grounded understanding of adsorption mechanisms at the molecular level.

A key advantage of the present approach lies in its ability to extract temperature-dependent thermodynamic parameters with high precision, thereby capturing the dynamic nature of adsorption processes. This capability allows the identification of systematic trends and structure–property relationships in porous materials, providing valuable insight into how surface chemistry and electronic structure govern adsorption behavior. In this context, the marked contrast observed between MgY and NH_4_Y highlights the sensitivity of the methodology to variations in surface composition and interaction mechanisms, confirming its effectiveness for the characterization of complex adsorption systems.

Furthermore, the identification of well-defined enthalpy–entropy compensation relationships for both dispersive and polar components demonstrates that even highly specific interactions obey underlying thermodynamic laws. This finding reinforces the internal consistency of the present framework and supports its applicability to a wide range of adsorbate–surface systems. Overall, the methodology provides a robust and unified platform for analyzing adsorption phenomena, bridging fundamental thermodynamic analysis with detailed molecular-level interpretation.

### 4.5. Separation Distance r(T) and Thermal Expansion Coefficient

**Figure 13:**
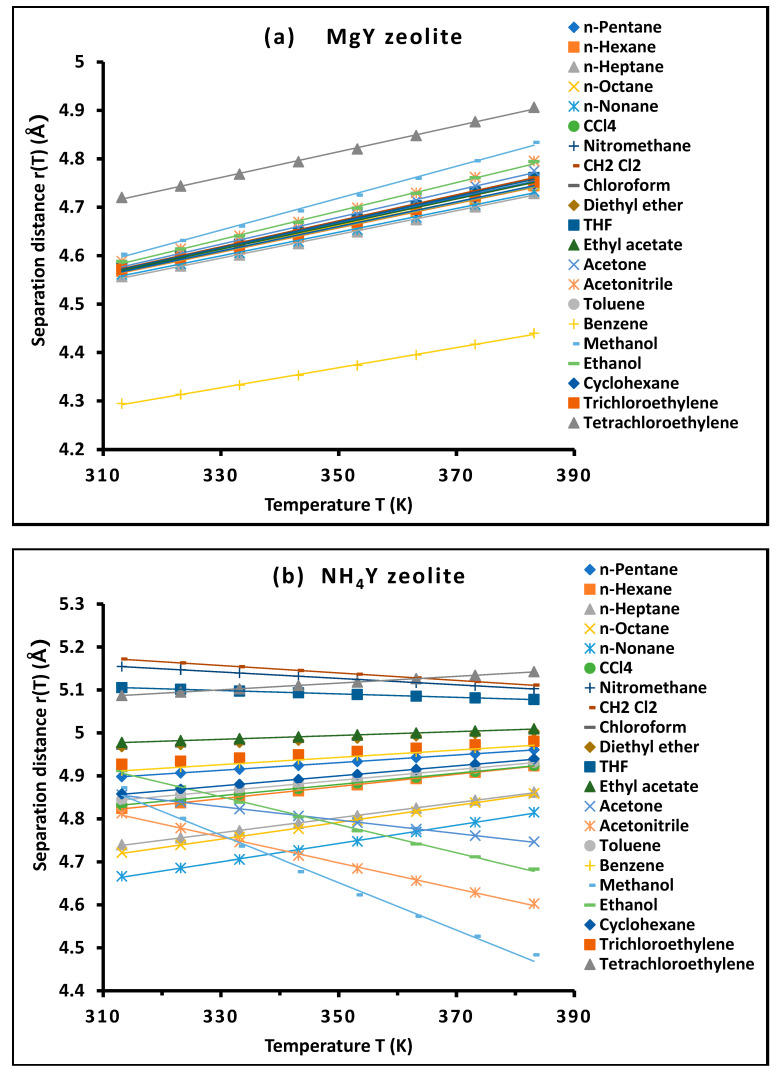
Temperature dependence of intermolecular separation distance. Linear variation in the adsorbate–surface separation distance, $r_{S-X}(T)$, for probe molecules on MgY and NH_4_Y zeolites. The slopes correspond to effective thermal expansion coefficients, while extrapolation to 0 K provides the intrinsic intermolecular distance $r_{0}$.

The temperature dependence of the intermolecular separation distance $r_{S-X}(T)$ between the adsorbed probe molecules and the zeolite surface was determined using the present thermodynamic framework. The calculated values are reported in Table S3, while their linear variations are illustrated in Figure 13 for both MgY and NH_4_Y materials.

The results provided in Table 3 reveal that, for all investigated solvent–solid pairs, the separation distance follows a remarkably linear dependence on temperature, which can be expressed by Equation (17):

(17) $$r_{S-X}\left(T\right)=r_{0}+\alpha_{\mathrm{eff}}\;T$$

where $r_{0}$ represents the extrapolated intermolecular distance at 0 K and $\alpha_{\mathrm{eff}}$ is an effective thermal expansion coefficient characteristic of the adsorbate–surface pair.

Within the present framework, the parameter $r_{0}$ represents a fundamental quantity corresponding to the equilibrium intermolecular distance at 0 K, where thermal motion is absent and interactions are purely governed by the intrinsic potential energy landscape.

The coefficient $\alpha_{\mathrm{eff}}$, in turn, can be interpreted as an effective thermodynamic expansion parameter, which integrates not only geometric effects but also the temperature dependence of interaction forces, including dispersion, induction, and acid–base contributions.

This interpretation goes beyond classical thermal expansion concepts and provides a direct link between thermodynamics and intermolecular forces, which is a distinctive feature of the present methodology.

The excellent linearity observed in Figure 13, with regression coefficients typically exceeding $R^{2}>0.998$, provides strong evidence for the validity of this formulation and confirms that the thermal evolution of intermolecular distances can be described within a simple yet physically meaningful linear model.

#### 4.5.1. Behavior of MgY: Structured and Uniform Expansion

For MgY, the values of $\alpha_{\mathrm{eff}}$ are consistently positive and lie within a narrow range, typically between $2.1\times 10^{-3}$ and $3.3\times 10^{-3}$ ÅK^−1^. This uniformity indicates that thermal expansion is governed by a well-defined interaction field, where the increase in temperature leads to a progressive and homogeneous increase in intermolecular separation.

The extrapolated distances $r_{0}$ for MgY fall within a relatively compact interval of 3.57 to 3.88 Å, depending on the nature of the probe molecule. These values are consistent with typical van der Waals contact distances and confirm that adsorption occurs within a short-range interaction regime dominated by dispersive and localized electrostatic contributions.

The slight variations observed among different probes reflect differences in molecular size, polarizability, and interaction strength. For instance, smaller or more strongly interacting molecules such as methanol and acetonitrile exhibit slightly lower $r_{0}$ values, indicating a closer approach to the surface.

Overall, MgY displays a highly structured and thermodynamically coherent adsorption environment, characterized by consistent thermal expansion behavior, a narrow distribution of intermolecular distances, and a strong coupling between temperature and interaction geometry, reflecting a well-organized and energetically stable interaction field.

#### 4.5.2. Behavior of NH_4_Y: Expanded and Flexible Interaction Field

In contrast, NH_4_Y exhibits significantly larger intermolecular distances, with $r_{0}$ values ranging from approximately 4.0 Å up to 6.6 Å, depending on the probe. This increase reflects a more diffuse interaction field, consistent with the presence of ammonium species and hydrogen-bonding interactions, which tend to occur at longer distances compared to purely dispersive contacts.

A striking feature of NH_4_Y is the wide variability of the thermal expansion coefficient $\alpha_{\mathrm{eff}}$, which spans both positive and negative values. While non-polar probes such as n-alkanes show small positive coefficients, several polar molecules—including acetonitrile, acetone, methanol, and ethanol—exhibit negative thermal expansion behavior.

This unusual phenomenon indicates that, for these molecules, the effective intermolecular distance decreases with increasing temperature, suggesting a thermally induced strengthening or reorganization of interactions. Such behavior may arise from enhanced alignment of dipolar molecules, restructuring of hydrogen-bonding networks, or deeper penetration of the adsorbate into the pore environment at elevated temperatures, reflecting a complex interplay between thermal activation and local interaction fields.

These results clearly demonstrate that NH_4_Y provides a highly flexible and adaptive adsorption environment, where the interaction geometry is strongly influenced by the nature of the probe molecule and the balance between dispersive and polar contributions.

#### 4.5.3. Comparison Between MgY and NH_4_Y

The comparison between the two zeolites highlights a fundamental difference in their adsorption behavior. MgY is characterized by short intermolecular distances and uniform positive thermal expansion, reflecting a rigid and well-defined interaction field dominated by Lewis acidity and dispersion forces. In contrast, NH_4_Y exhibits larger separation distances and variable, sometimes negative, thermal expansion coefficients, indicating a softer, more flexible, and structurally responsive interaction landscape governed by hydrogen bonding and polar interactions. This contrast provides direct molecular-level evidence of the distinct surface chemistries and adsorption mechanisms of the two materials.

#### 4.5.4. Methodological Significance and Novelty

The determination of temperature-dependent intermolecular distances from IGC data represents a major advancement in surface thermodynamics. Unlike conventional approaches, in which interaction distances are typically treated as fixed or empirically estimated parameters, the present methodology provides direct experimental access to r(T), enables the determination of the extrapolated distance $r_{0}$ at 0 K, allows quantification of thermal expansion at the molecular scale, and reveals non-classical behaviors such as negative expansion, thereby offering a more physically grounded description of adsorption geometry.

This capability provides a unique bridge between macroscopic thermodynamic measurements and microscopic interaction geometry, offering unprecedented insight into adsorption phenomena.

### 4.6. Molecular Surface Area

A major outcome of the present study is the thermodynamic determination of the molecular surface area of adsorbed solvents, $a_{X/S}(T)$, as a function of temperature. In classical inverse gas chromatography analyses, the molecular surface area of probe molecules is generally treated as a fixed geometric parameter derived from molecular models. However, such an assumption neglects the fundamental role of adsorbate–surface interactions, which inherently modify the effective contact area during adsorption.

**Figure 14:**
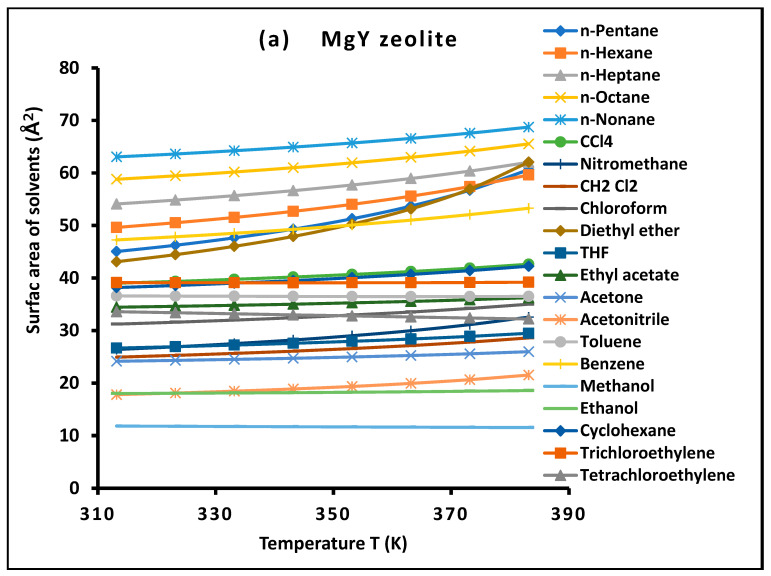

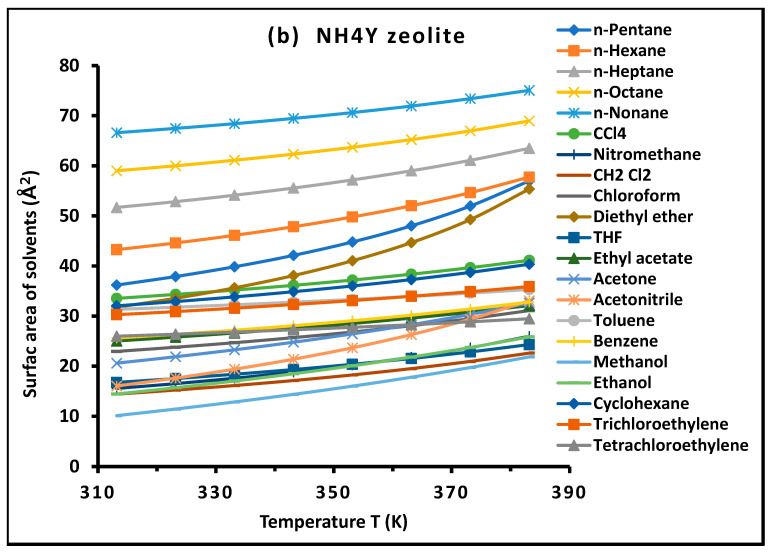
**Temperature dependence of the molecular surface area of adsorbed probes.** Variation in the effective molecular surface area, $a_{X/S}(T)$, of probe molecules adsorbed on MgY and NH_4_Y zeolites. The results demonstrate that the adsorption cross-section is a thermodynamic quantity that evolves with temperature and interaction strength.

The present results clearly demonstrate that the molecular surface area is not an intrinsic constant, but rather a thermodynamic quantity that depends on both temperature and the nature of the solid–adsorbate interaction. The values reported in Table S6 and illustrated in Figure 14 show that $a_{X/S}(T)$ varies systematically with temperature for all investigated solvents on both MgY and NH_4_Y zeolites.

#### 4.6.1. Linear Temperature Dependence

As a first approximation, the variation in the molecular surface area can be described by the linear Relation (18):

(18) $$a_{X/S}(T)=a_{X/S}(298.15)+\lambda_{X/S}(T-298.15)\;$$

where $\lambda_{X/S}$ given in Table 4 represents an effective thermal expansion coefficient of the molecular surface area, reflecting the sensitivity of the adsorption cross-section to temperature.

For MgY, the values of $\lambda_{X/S}$ are generally positive for most probes, indicating that the effective surface area increases with temperature. This behavior is consistent with a gradual weakening of intermolecular interactions, leading to a slight expansion of the adsorbate footprint on the surface. The magnitude of this effect depends on the nature of the probe, with larger and more flexible molecules such as n-alkanes exhibiting higher expansion coefficients.

In contrast, several probes on MgY—such as toluene, methanol, and tetrachloroethylene—exhibit very small or even negative values of $\lambda_{X/S}$, indicating a reduction in the effective surface area with increasing temperature. This non-classical behavior suggests that adsorption is accompanied by reorganization effects, possibly involving changes in molecular orientation or interaction strength.

For NH_4_Y, the thermal expansion coefficients are generally larger than those observed for MgY, reflecting a more pronounced sensitivity of the adsorption geometry to temperature. This is consistent with the more flexible and polar nature of the NH_4_Y surface, where hydrogen bonding and dipolar interactions can significantly influence the effective contact area.

Overall, the linear model provides a reasonable first-order description of the data, with coefficients of determination typically around $R^{2}\approx 0.98$, although notable deviations are observed in specific cases (e.g., trichloroethylene on MgY), indicating the need for a more refined description.

#### 4.6.2. Quadratic Temperature Dependence: A More Accurate Description

A more detailed analysis reveals that the variation of $a_{X/S}(T)$ is more accurately described by a **quadratic dependence on temperature**:

$$a_{X/S}(T)=a_{X/S}(T_{0})+\lambda_{1}(T-T_{0})+\lambda_{2}(T-T_{0}{)}^{2}$$

where $\lambda_{1}$ and $\lambda_{2}$ are coefficients reflecting first- and second-order temperature effects.

The results presented in Table 5 demonstrate that this quadratic formulation reproduces the experimental data with **remarkable accuracy**, with regression coefficients generally exceeding $R^{2}>0.999$ for most solvent–solid systems. This clearly indicates that the molecular surface area is governed by **non-linear thermodynamic effects**, which cannot be captured by a simple linear approximation.

The quadratic term $\lambda_{2}$ captures the progressive modification of intermolecular interactions with temperature, reflecting changes in molecular mobility, variations in adsorption geometry, and the evolving balance between dispersive and polar interaction contributions.

This behavior reflects the fact that adsorption occurs within a **dynamic molecular environment**, where the effective contact area is continuously adjusted as a function of temperature.

#### 4.6.3. Comparison Between MgY and NH_4_Y

The comparison between MgY and NH_4_Y reveals significant differences in the behavior of the molecular surface area.

For MgY, the values of $a_{X/S}(298.15)$ are generally larger and exhibit a relatively moderate temperature dependence. This indicates that adsorption occurs on a structured and relatively rigid surface, where the geometry of interaction is well-defined and only weakly perturbed by temperature.

In contrast, NH_4_Y shows smaller initial surface areas for many probes but significantly larger temperature-dependent variations. This behavior reflects a more flexible and adaptive adsorption environment, where the effective molecular footprint is strongly influenced by temperature and by the nature of polar interactions.

In particular, the stronger temperature dependence observed for polar probes on NH_4_Y suggests that hydrogen bonding and dipolar interactions play a major role in determining the adsorption geometry, leading to enhanced sensitivity of the surface area to thermal effects.

#### 4.6.4. Physical Interpretation and Implications

The results obtained in this work fundamentally revise the classical concept of molecular surface area in adsorption studies. Rather than being a purely geometric parameter, $a_{X/S}(T)$ must be regarded as a **thermodynamic quantity**, reflecting the balance between intermolecular forces and thermal motion.

The temperature dependence of the molecular surface area provides direct insight into the strength and nature of adsorbate–surface interactions, the flexibility of the adsorption environment, and the extent of molecular reorganization occurring upon adsorption. In this context, the observed non-linear behavior highlights the importance of considering **higher-order thermodynamic effects** when analyzing adsorption phenomena.

#### 4.6.5. Methodological Significance and Novelty

The determination of a temperature-dependent molecular surface area represents a major conceptual and methodological advancement. The present approach challenges the conventional assumption of a constant molecular area, introduces a direct thermodynamic framework for quantifying adsorption cross-sections, reveals the inherently dynamic nature of adsorption at the molecular scale, and establishes a robust quantitative relationship between surface energetics and adsorption geometry.

This methodology offers a powerful new framework for the interpretation of IGC data and opens the way for a more rigorous and physically meaningful characterization of solid surfaces.

### 4.7. London Dispersive Surface Energy

The London dispersive component of the surface energy, $\gamma_{s}^{d}(T)$, of MgY and NH_4_Y zeolites was determined using the Hamieh methodology, which is based on the Hamaker constant $A_{12}$ of the solid–n-alkane system. This approach provides a rigorous thermodynamic framework that directly links macroscopic surface energy to molecular-scale dispersion interactions.

**Figure 15:**
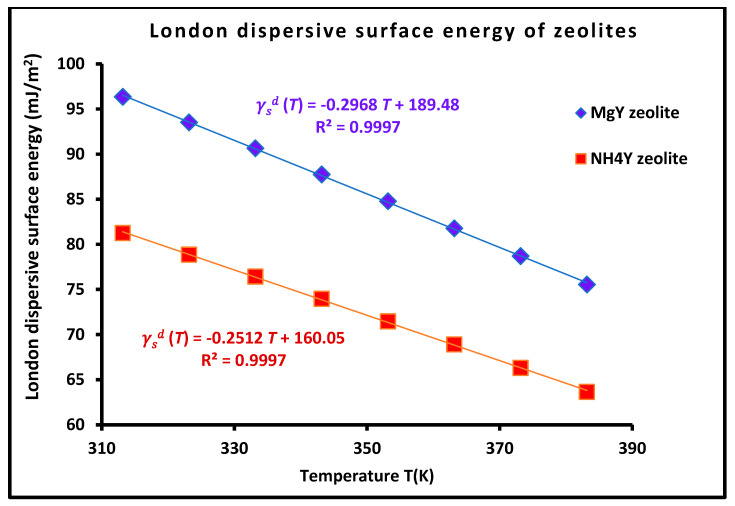
Evolution of the London dispersive surface energy $\gamma_{s}^{d}$ of MgY and NH_4_Y as a function of the temperature.

The calculated values of $\gamma_{s}^{d}(T)$ are reported in Table S7, while their temperature dependence is illustrated in Figure 15.

The results plotted in Figure 15 and given in Table 6 reveal a remarkably linear decrease in the London dispersive surface energy with increasing temperature for both zeolites, described by the following relations:

$$\mathrm{MgY}:\;\gamma_{s}^{d}(T)=-0.297\;T+189.48\;{\mathrm{NH}}_{4}Y:\;\gamma_{s}^{d}(T)=-0.251\;T+160.05$$

The excellent linearity, with regression coefficients $R^{2}=0.9997$, confirms the robustness of the present methodology and demonstrates that the temperature dependence of dispersive surface energy can be accurately captured within a first-order thermodynamic framework.

#### 4.7.1. Magnitude and Surface Character

A first key observation concerns the higher values of $\gamma_{s}^{d}$ for MgY compared to NH_4_Y over the entire temperature range. The extrapolated values at 0 K are:

- $\gamma_{s}^{d}(0\;K)=189.48\;{\mathrm{mJ}\;m}^{-2}$ for MgY;
- $\gamma_{s}^{d}(0\;K)=160.05\;{\mathrm{mJ}\;m}^{-2}$ for NH_4_Y.

This difference reflects a stronger dispersive interaction field in MgY, which can be attributed to the presence of Mg^2+^ cations inducing enhanced electronic polarization of the adsorbed molecules. In contrast, the lower dispersive surface energy of NH_4_Y indicates a less polarizable surface, consistent with the presence of ammonium species and the dominance of polar (non-dispersive) interactions.

#### 4.7.2. Temperature Dependence and Surface Entropy

The negative slopes of the linear relationships correspond to the London dispersive surface entropy, defined as:

$$\varepsilon_{s}^{d}=\frac{d\gamma_{s}^{d}}{dT}$$

The obtained values are:

- $\varepsilon_{s}^{d}=-0.297\;{\mathrm{mJ}\;m}^{-2}{\;K}^{-1}$ for MgY;
- $\varepsilon_{s}^{d}=-0.251\;{\mathrm{mJ}\;m}^{-2}{\;K}^{-1}$ for NH_4_Y.

The negative sign indicates that the dispersive surface energy decreases with increasing temperature, reflecting the progressive weakening of London interactions due to increased thermal motion and reduced correlation between fluctuating dipoles.

The larger magnitude of $\varepsilon_{s}^{d}$ for MgY suggests a stronger sensitivity of its dispersive interactions to temperature, which is consistent with its higher initial surface energy and more structured interaction field.

#### 4.7.3. Intrinsic Maximum Temperature

An important parameter derived from the present analysis is the **intrinsic maximum temperature**, $T_{\max}$, at which the dispersive surface energy extrapolates to zero:

- $T_{\max}=637.98\;K$ for MgY;
- $T_{\max}=637.65\;K$ for NH_4_Y.

The close similarity of these values for both zeolites suggests that, despite differences in magnitude, the thermal stability of dispersive interactions is governed by a common physical mechanism, likely related to the universal temperature dependence of electronic polarization.

This result is particularly significant, as it indicates that the temperature at which dispersive interactions vanish is intrinsic to the nature of London forces, rather than to the specific chemical composition of the surface.

#### 4.7.4. Comparison Between MgY and NH_4_Y

The comparison between the two zeolites reveals a clear distinction in their dispersive behavior. MgY exhibits higher dispersive surface energy and a stronger temperature dependence, reflecting a more polarizable and energetically structured surface. In contrast, NH_4_Y shows lower dispersive energy and weaker sensitivity to temperature, consistent with a surface where polar and hydrogen-bonding interactions play a dominant role over dispersion forces.

This distinction is fully consistent with the trends observed in the polar adsorption parameters and confirms the complementarity between dispersive and specific interaction contributions.

#### 4.7.5. Methodological Significance and Novelty

The determination of $\gamma_{s}^{d}(T)$ using the Hamaker constant represents a major advancement in surface characterization. Unlike conventional IGC methods, which rely primarily on empirical correlations, the present approach is firmly grounded in fundamental London dispersion theory, explicitly incorporates temperature-dependent intermolecular distances, and establishes a direct and physically consistent link between microscopic interaction parameters and macroscopic surface energy.

This methodology allows, for the first time, a quantitative and physically consistent determination of dispersive surface energy as a function of temperature, offering new insights into the thermodynamic behavior of solid surfaces.

It is important to emphasize that the linear temperature dependence of the adsorption free energy reflects classical thermodynamic behavior, whereas the Lewis acid–base modeling developed in the present work is intrinsically nonlinear. The five-parameter formulation introduces higher-order terms and coupling effects, leading to a nonlinear dependence of adsorption energy on donor and acceptor properties, which cannot be captured by conventional linear models.

This distinction highlights a fundamental limitation of classical approaches, which assume independent and additive contributions of acid and base interactions. In contrast, the present framework accounts for the intrinsic coupling between donor and acceptor effects, as well as their nonlinear contributions, providing a more realistic and physically consistent description of adsorption on heterogeneous surfaces.

On this basis, the detailed analysis of Lewis acid–base interactions is presented in Section 4.8, where the thermodynamic parameters governing donor–acceptor behavior are quantitatively determined and discussed for MgY and NH_4_Y zeolites.

### 4.8. Lewis Acid–Base Parameters of Zeolite Surfaces

The use of highly parameterized models in the present work is not merely a mathematical refinement but reflects the intrinsic complexity of Lewis acid–base interactions at heterogeneous surfaces. Classical approaches based on two or three parameters assume that donor and acceptor contributions are independent and linearly additive. However, adsorption on real surfaces such as zeolites involves multiple interaction mechanisms, including polarization, charge transfer, hydrogen bonding, and cooperative effects between neighboring sites. These phenomena inherently introduce nonlinearities and coupling between donor and acceptor properties.

The five-parameter model employed in this study explicitly accounts for these effects through the inclusion of an amphoteric coupling term and second-order contributions. The parameter K describes the simultaneous interaction between donor and acceptor characteristics, while $K_{2A}$ and $K_{2D}$ capture curvature effects associated with nonlinear donor–acceptor behavior. The necessity of these additional terms is quantitatively demonstrated by the statistical analysis, which shows that simpler models systematically fail to reproduce the experimental data with comparable accuracy. In contrast, the five-parameter formulation provides significantly improved fits, as evidenced by higher coefficients of determination and lower RMSE, AIC, and BIC values.

Importantly, each additional parameter introduced in the model has a clear physical interpretation and is directly linked to a specific interaction mechanism. Therefore, the increased number of parameters does not represent overfitting but rather a more complete and physically meaningful description of adsorption energetics. This result confirms that Lewis acid–base interactions on complex surfaces cannot be adequately described by simplified linear models and require a framework capable of capturing their intrinsic nonlinearity and cooperative nature.

#### 4.8.1. Comparative Statistical Evaluation of the Adsorption Models

The thermodynamic characterization of Lewis acid–base interactions on MgY and NH_4_Y zeolites was carried out using a hierarchy of models of increasing complexity, ranging from the classical two-parameter (2-P) formulation to the extended five-parameter Hamieh model (5-P). Intermediate formulations, including the three-parameter (3-P) and two four-parameter variants (4.2-P and 4.3-P), were also systematically evaluated.

A rigorous statistical analysis was performed to assess the ability of each model to describe the experimental adsorption data. The evaluation relied on complementary statistical indicators, namely the coefficient of determination $R^{2}$, the root mean square error (RMSE), and the information criteria AIC and BIC, which account for both goodness of fit and model complexity. To enable direct comparison, normalized forms of these indicators were combined into a global score reflecting the overall performance of each model.

The results summarized in Table 7 clearly demonstrate that, for both MgY and NH_4_Y, the five-parameter model systematically outperforms all simpler formulations. This conclusion is supported by the highest $R^{2}$ values ($R^{2}=0.9862$ for both materials), the lowest RMSE values (2.25 for MgY and 4.45 for NH_4_Y), and the minimum AIC and BIC values, collectively indicating an optimal balance between model accuracy and complexity.

In contrast, the classical 2-P and 3-P models, although capturing general trends, fail to adequately describe the complexity of the adsorption behavior. The intermediate 4-P models improve the fit but remain insufficient, as evidenced by higher residual errors and less favorable information criteria.

The global scoring approach further confirms this conclusion, assigning a maximum score of 1.0000 to the five-parameter model for both zeolites. This result provides strong statistical evidence that the inclusion of higher-order interaction terms is essential for an accurate thermodynamic description of Lewis acid–base adsorption.

#### 4.8.2. Validation of the Five-Parameter Model

The superiority of the five-parameter model reflects the intrinsic complexity of acid–base interactions at the zeolite surface. Unlike simpler models, which assume linear and independent donor–acceptor contributions, the 5-P formulation explicitly incorporates amphoteric coupling effects through the parameter K, along with nonlinear donor and acceptor interactions described by $K_{2D}$ and $K_{2A}$, thereby providing a more comprehensive and physically realistic representation of surface interactions.

These additional terms enable the model to capture deviations from ideal linear behavior, which arise from cooperative effects, site heterogeneity, and the interplay between dispersive and specific interactions.

The statistical validation presented in Table 8 demonstrates that such nonlinear contributions are not negligible but rather fundamental to the adsorption mechanism, particularly for complex surfaces such as zeolites.

#### 4.8.3. Quantitative Analysis of Lewis Acidity and Basicity

The thermodynamic parameters obtained from the five-parameter model (Table 9) reveal significant differences between MgY and NH_4_Y.

For MgY, the five-parameter model yields:

- $K_{A}=-0.2250$;
- $K_{D}=0.7231$;
- $K_{D}/K_{A}=-3.2141$;
- $K_{A}+K_{D}=0.4981$.

The relatively high value of $K_{D}$ indicates that MgY exhibits a strong Lewis basic character, while the negative value of $K_{A}$ suggests a complex or non-classical acidic contribution. This behavior can be interpreted as the result of strong polarization effects induced by Mg^2+^ cations, which enhance electron donor interactions while modifying the effective acidity of the surface.

The presence of non-zero higher-order parameters ($K=7.8\times 10^{-4}$, $K_{2A}=0.0073$, $K_{2D}=-0.0088$) confirms the importance of nonlinear effects, indicating that adsorption cannot be described by simple additive contributions.

For NH_4_Y, the five-parameter model provides:

- $K_{A}=-0.2879$;
- $K_{D}=1.3413$;
- $K_{D}/K_{A}=-4.6581$;
- $K_{A}+K_{D}=1.0533$.

The significantly higher value of $K_{D}$ for NH_4_Y compared to MgY indicates a much stronger electron donor (basic) character, which may at first appear counterintuitive considering its protonic nature. However, this behavior reflects the complex interplay between protonated ammonium species, hydrogen-bonding interactions, and enhanced polarization effects within the zeolite framework, all of which contribute to stabilizing electron donor interactions at the surface.

The larger value of $K_{A}+K_{D}$ further indicates a **higher overall polarity** of NH_4_Y, consistent with its stronger interaction with polar probe molecules observed in Section 4.3.

The higher-order parameters ($K_{2A}=0.0105$, $K_{2D}=-0.0208$) are also more pronounced than in MgY, highlighting a stronger degree of nonlinearity and interaction coupling.

#### 4.8.4. Physical Interpretation of Nonlinear Parameters

The introduction of the parameters K, $K_{2A}$, and $K_{2D}$ provides deeper insight into the nature of adsorption processes. The parameter K reflects amphoteric coupling, corresponding to the simultaneous involvement of donor and acceptor interactions, whereas $K_{2A}$ and $K_{2D}$ account for second-order curvature effects, highlighting deviations from linear adsorption behavior and capturing the intrinsic nonlinearity of surface interactions.

The positive values of $K_{2A}$ indicate an enhancement of acceptor interactions at higher interaction strengths, whereas the negative values of $K_{2D}$ suggest a saturation or weakening of donor interactions beyond a certain level.

These effects are particularly pronounced for NH_4_Y, confirming that adsorption on this material involves cooperative and highly non-linear mechanisms, likely associated with hydrogen bonding networks and site heterogeneity.

#### 4.8.5. Comparison Between MgY and NH_4_Y

The comparative analysis of MgY and NH_4_Y zeolites reveals a clear and fundamental distinction in their Lewis acid–base behavior, reflecting differences in surface chemistry and interaction mechanisms.

MgY is characterized by a moderate polarity and a relatively well-structured interaction field, where adsorption is predominantly governed by polarization effects and classical Lewis-type interactions. The thermodynamic behavior remains relatively regular, with limited nonlinear contributions, indicating that adsorption occurs on energetically well-defined sites with comparatively uniform interaction strengths.

In contrast, NH_4_Y exhibits a significantly higher polarity and a markedly stronger electron donor character, accompanied by pronounced nonlinear effects. These features indicate a more complex interaction landscape, in which adsorption is strongly influenced by hydrogen bonding, protonic interactions, and cooperative phenomena. The enhanced nonlinearity reflects the presence of coupled interaction mechanisms and a higher degree of surface heterogeneity.

This fundamental distinction between MgY and NH_4_Y is fully consistent with the trends previously observed in dispersive surface energy, polar thermodynamic parameters, and temperature-dependent intermolecular distances. Altogether, these results demonstrate the strong internal coherence of the present analysis and confirm that the proposed thermodynamic framework provides a unified and physically meaningful description of adsorption on zeolite surfaces.

#### 4.8.6. Methodological Significance and General Implications

The results presented here provide strong evidence that classical linear models are insufficient to adequately describe Lewis acid–base interactions on complex surfaces. In contrast, the five-parameter Hamieh model offers a comprehensive and physically meaningful framework that captures nonlinear donor–acceptor interactions, accounts for amphoteric coupling effects, and reflects the intrinsic heterogeneity of real surfaces.

This work therefore establishes the five-parameter model as a universal and statistically validated approach for the thermodynamic characterization of solid surfaces.

### 4.9. Comparative Analysis with Previous IGC Studies

The interpretation of the present results must be considered in the context of limitations inherent to classical inverse gas chromatography methodologies. Many widely used approaches assume a constant molecular surface area of probe molecules and neglect their temperature dependence. However, recent thermodynamic analyses have demonstrated that the molecular surface area is not a fixed geometric parameter but varies with temperature and interaction strength. Neglecting this effect leads to systematic errors in the determination of dispersive surface energy.

Similarly, conventional procedures for separating dispersive and polar contributions often rely on simplified linear extrapolations, which may not accurately capture the complexity of donor–acceptor interactions. This can result in significant deviations in the calculated Lewis acid–base parameters, particularly for heterogeneous surfaces such as zeolites.

In contrast, the present methodology provides a thermodynamically consistent framework in which both intermolecular distance and molecular surface area are treated as temperature-dependent variables, and dispersive and specific contributions are rigorously separated. This approach not only improves the quantitative accuracy of the derived parameters but also provides a more realistic description of adsorption at the molecular level.

A meaningful assessment of the present results can be achieved through comparison with the work of Bilgiç and Tümsek [38], who investigated the same MgY and NH_4_Y zeolites using conventional inverse gas chromatography methodologies. While both studies aim to characterize the thermodynamic properties and acid–base behavior of these zeolitic materials, the conceptual frameworks and resulting interpretations exhibit substantial differences.

In the classical approach adopted by Bilgiç and Tümsek, adsorption thermodynamic parameters were derived from retention data under the assumption of a temperature-independent interaction distance and a uniform dispersive surface energy. The dispersive component of the surface energy was evaluated using traditional models such as the Dorris–Gray method, while the acid–base properties were interpreted within the Gutmann donor–acceptor framework. Although these approaches provide a first-order description of surface energetics, they inherently rely on simplified assumptions that do not fully capture the molecular complexity of adsorption on microporous solids.

In contrast, the present work introduces a thermodynamically consistent framework in which the intermolecular distance between the adsorbate and the surface is explicitly treated as a temperature-dependent quantity, r(T). This fundamental advancement allows a direct coupling between adsorption energy, molecular separation, and thermal effects, leading to a more physically grounded interpretation of both dispersive and polar contributions. The excellent linearity observed for $\Delta G_{a}^{d}(T)$ and $\Delta G_{a}^{p}(T)$, together with the determination of well-defined enthalpy–entropy compensation temperatures, confirms the internal consistency of this approach and its ability to describe adsorption within the Henry regime.

A key point of divergence lies in the evaluation of the London dispersive surface energy. While the classical methodology yields surface energy values that are often treated as intrinsic constants, the present analysis demonstrates that $\gamma_{s}^{d}$ is intrinsically temperature-dependent, decreasing linearly with increasing temperature. This behavior is quantitatively linked to the Hamaker constant-based formalism, which provides a direct connection between macroscopic surface energy and microscopic intermolecular interactions. As a result, the present values of $\gamma_{s}^{d}(T)$ not only exhibit superior physical meaning but also enable the determination of additional quantities such as the surface entropy and the intrinsic maximum temperature $T_{\mathrm{Max}}$—parameters that are inaccessible within classical treatments.

Significant differences are also observed in the description of Lewis acid–base interactions. In the work of Bilgiç and Tümsek, the surface is characterized by global acidity and basicity constants derived from linear correlations. However, the present study demonstrates that such a description is insufficient to account for the complexity of real adsorption systems. The application of the Hamieh five-parameter model reveals the importance of nonlinear donor–acceptor interactions and amphoteric coupling, as evidenced by the superior statistical performance (higher $R^{2}$, lower AIC, BIC, and RMSE). This extended model provides a richer and more accurate representation of the interaction landscape, particularly for NH_4_Y, where strong polarity and cooperative effects dominate.

Furthermore, the introduction of the temperature-dependent intermolecular distance r(T) provides new insight into the structural aspects of adsorption. The observed linear behavior and the determination of the effective thermal expansion coefficient $\alpha_{\mathrm{eff}}$ highlight the dynamic nature of the adsorbed phase, in contrast with the static picture implicitly assumed in classical IGC analyses. The markedly different trends obtained for MgY and NH_4_Y further emphasize the role of surface composition and polarity in governing adsorption geometry.

Overall, the comparison clearly demonstrates that, although the classical study of Bilgiç and Tümsek provides useful qualitative information, it remains limited by its simplifying assumptions and empirical formulations. The present work, by contrast, offers a unified and physically consistent thermodynamic description that integrates dispersive, polar, and structural effects within a single framework. This comprehensive approach not only enhances the accuracy of the extracted parameters but also provides deeper insight into the molecular mechanisms governing adsorption on zeolitic surfaces.

### 4.10. From Analysis to Application: Implications for Adsorption Engineering and Material Design

The comprehensive thermodynamic framework developed in the present work provides not only a refined description of adsorption phenomena on zeolitic surfaces, but also establishes a direct and quantitative bridge between fundamental physicochemical analysis and practical engineering applications. This transition from analysis to application is enabled by the explicit determination of temperature-dependent energetic, structural, and molecular interaction parameters, which together define a predictive platform for adsorption-driven processes.

A central outcome of this study is the demonstration that adsorption cannot be adequately described by static surface properties. Instead, the results clearly show that key quantities such as the London dispersive surface energy $\gamma_{s}^{d}(T)$ and the intermolecular distance r(T) evolve continuously with temperature. This finding has immediate implications for process design, as it indicates that adsorption performance is intrinsically temperature-sensitive, even within relatively narrow operating ranges. In practical terms, this means that adsorption selectivity, retention behavior, and surface affinity cannot be reliably predicted using temperature-independent parameters, as is commonly assumed in classical approaches.

The linear decrease of $\gamma_{s}^{d}(T)$ with temperature reflects a progressive weakening of dispersive interactions, which directly affects the adsorption capacity and selectivity toward non-polar molecules. For MgY, the higher intrinsic dispersive surface energy and its stronger temperature sensitivity suggest a material that is particularly suitable for hydrocarbon separation and non-polar adsorption processes, especially under controlled thermal conditions. In contrast, NH_4_Y, with its lower dispersive energy but significantly stronger polar contribution, is more appropriate for polar molecule adsorption, hydrogen-bonding interactions, and selective capture of oxygenated or nitrogen-containing compounds.

The introduction of the temperature-dependent intermolecular distance r(T) represents a major advancement for adsorption engineering. The determination of the effective thermal expansion coefficient $\alpha_{\mathrm{eff}}$ provides direct insight into the mechanical and structural response of the adsorbed phase, revealing that adsorption is accompanied by a measurable expansion or contraction at the molecular scale. This effect becomes particularly significant for polar probes on NH_4_Y, where negative or weakly positive values of $\alpha_{\mathrm{eff}}$ indicate the presence of strong localized interactions and constrained adsorption geometries. Such information is essential for designing materials with tailored pore environments and controlled confinement effects.

Another critical contribution of the present work lies in the rigorous characterization of enthalpy–entropy compensation phenomena for both dispersive and polar adsorption. The identification of well-defined compensation temperatures $T_{\mathrm{int}}$ provides a thermodynamic criterion for distinguishing between adsorption regimes dominated by enthalpic stabilization and those governed by entropic effects. From an application standpoint, this allows the optimization of operating conditions by selecting temperature ranges where adsorption is maximized or selectively enhanced for specific classes of molecules.

The application of the **Hamieh five-parameter model** further strengthens the transition toward practical applications by offering a highly accurate and physically meaningful description of Lewis acid–base interactions. Unlike classical linear models, the present approach accounts for **nonlinear donor–acceptor coupling and higher-order interaction terms**, which are particularly relevant in heterogeneous and highly polar systems. The clear distinction observed between MgY and NH_4_Y demonstrates that surface functionality can be finely tuned to target specific adsorption mechanisms, ranging from weak polarization interactions to strong hydrogen bonding and cooperative effects.

From an industrial perspective, these findings have direct implications for a wide range of applications, including:

- **Gas separation and purification**, where accurate prediction of adsorption selectivity is critical for process efficiency;
- **Catalysis**, where the acid–base properties of the support influence reaction pathways and product distribution;
- **Environmental remediation**, particularly in the adsorption of volatile organic compounds (VOCs) and toxic polar species;
- **Chromatographic separations**, where retention behavior is governed by subtle differences in intermolecular interactions;
- **Energy-related applications**, such as CO_2_ capture and storage, where temperature-dependent adsorption plays a key role.

Importantly, the present framework enables not only the interpretation of experimental data but also the prediction of adsorption behavior under varying thermodynamic conditions, thereby providing a powerful tool for material screening and optimization. The ability to relate macroscopic observables, such as retention volumes and surface energies, to microscopic quantities, including intermolecular distance and interaction potentials, represents a significant step toward the rational design of advanced adsorbent materials.

Furthermore, the internal coherence observed across all measured quantities—dispersive energy, polar thermodynamics, intermolecular distance, and Lewis acid–base parameters—confirms the robustness of the proposed methodology. This unified description demonstrates that adsorption on zeolitic surfaces can be understood within a consistent thermodynamic framework that integrates energetic, structural, and molecular aspects. Such an approach is essential for advancing the field from empirical characterization toward predictive and application-oriented surface science.

From an industrial and technological standpoint, the present framework opens new perspectives for the rational design of adsorbent materials and processes. By linking macroscopic observables—such as retention volumes and surface energies—to microscopic quantities including intermolecular distances, interaction potentials, and Hamaker-based dispersive forces, the methodology enables a predictive and scalable approach to adsorption engineering. This capability is particularly relevant for emerging challenges in sustainable chemical processes, where precise control of molecular interactions is required to enhance selectivity, reduce energy consumption, and improve process efficiency. Furthermore, the integration of temperature-dependent thermodynamic parameters into adsorption models provides a robust foundation for coupling experimental characterization with computational approaches, including molecular simulation and process modeling. In this context, the present work contributes to the development of next-generation adsorption technologies, bridging the gap between fundamental surface science and real-world applications, in full alignment with the concept of “From Analysis to Application.”

### 4.11. Synergistic Contribution of Experimental and Theoretical Approaches to the Elucidation of Adsorption Mechanisms

A central objective of the present work is to establish a rigorous and physically consistent link between experimentally measured adsorption data and the underlying molecular mechanisms governing probe–surface interactions. This is achieved through the combined use of inverse gas chromatography at infinite dilution (IGC-ID) and an advanced thermodynamic framework integrating a Hamaker-based dispersive formalism with an extended five-parameter Lewis acid–base model.

#### 4.11.1. Contribution of Experimental Measurements

The IGC-ID experiments provide direct access to fundamental thermodynamic observables, namely the net retention volumes and their temperature dependence, from which the adsorption free energy $\Delta G_{a}^{0}(T)$ is derived. Owing to the infinite dilution regime, probe–probe interactions are negligible, and the measured quantities reflect exclusively probe–surface interactions.

From these measurements, primary thermodynamic parameters—including dispersive and polar components of the adsorption free energy, as well as the corresponding enthalpies and entropies—are obtained with high precision. The excellent linearity of $\Delta G_{a}(T)$ versus temperature (R^2^ ≈ 1) confirms that adsorption occurs in the Henry regime and validates the reliability of the experimental dataset.

However, while these measurements provide robust thermodynamic quantities, they do not by themselves yield direct insight into the microscopic origin of the interactions or the role of molecular and electronic structure.

#### 4.11.2. Contribution of Theoretical Modeling

The theoretical framework developed in this work enables a quantitative interpretation of the experimental data in terms of fundamental interaction parameters.

First, the Hamaker-based formulation establishes a direct relationship between the dispersive free energy and intrinsic electronic properties such as deformation polarizability, ionization energy, and intermolecular separation distance. This approach allows the determination of temperature-dependent intermolecular distances r(T), London potential parameters, and dispersive surface energy $\gamma_{s}^{d}(T)$, thereby providing a molecular-scale description of dispersive interactions.

Second, the five-parameter Lewis acid–base model extends classical approaches by incorporating amphoteric coupling and nonlinear donor–acceptor effects. Through the parameters $K_{A}$, $K_{D}$, K, $K_{2A}$, and $K_{2D}$, the model captures the intrinsic complexity of acid–base interactions on heterogeneous surfaces such as zeolites.

Unlike conventional linear models, this formulation allows a rigorous separation of dispersive and specific contributions and accounts for higher-order interaction effects, leading to a more accurate and physically meaningful representation of adsorption energetics.

#### 4.11.3. Integrated Interpretation: From Thermodynamic Data to Adsorption Mechanisms

The combined use of experimental measurements and theoretical modeling provides a powerful tool for refining the understanding of adsorption mechanisms.

The experimental data define the thermodynamic landscape of adsorption, while the theoretical framework translates these macroscopic quantities into microscopic interaction parameters. This integration enables the identification of the dominant interaction types (dispersion, polarization, hydrogen bonding), the quantification of their relative contributions, and the characterization of the spatial and energetic organization of adsorption sites.

For MgY, the high dispersive enthalpies, narrow distribution of compensation temperatures, and strong temperature dependence of surface energy collectively indicate a structured adsorption environment dominated by polarization-enhanced London interactions associated with Mg^2+^ cations. The theoretical analysis confirms that these effects originate from the high charge density and strong local electric fields, which increase the effective polarizability of the adsorbate–surface system.

In contrast, NH_4_Y exhibits lower dispersive energies, broader distributions of thermodynamic parameters, and non-classical behaviors for several polar probes. These features reveal a more heterogeneous and dynamically responsive adsorption environment, where hydrogen bonding and proton-mediated interactions play a major role. The theoretical model captures these effects through enhanced nonlinear contributions and weaker dispersive coupling.

#### 4.11.4. Advancement Beyond Classical Approaches

The integration of experimental and theoretical approaches in the present work represents a significant advancement over conventional IGC methodologies. Classical treatments typically provide empirical surface energy values without explicitly linking them to molecular-scale interaction mechanisms.

In contrast, the present methodology establishes a direct connection between measurable thermodynamic quantities and fundamental electronic properties, incorporates temperature-dependent structural parameters such as intermolecular distance and molecular surface area, and provides a unified framework capable of describing both linear and nonlinear adsorption phenomena.

As a result, adsorption is no longer treated as a static process but as a dynamic, temperature-dependent phenomenon governed by the coupled evolution of energetic and structural parameters.

#### 4.11.5. Implications for Surface Characterization and Material Design

This integrated approach significantly enhances the predictive power of adsorption analysis. By linking macroscopic observables to microscopic interaction parameters, it enables the rational interpretation of adsorption behavior across different materials and probe molecules.

Furthermore, the ability to distinguish between structured and heterogeneous adsorption environments, as demonstrated for MgY and NH_4_Y, provides valuable insight for the design and optimization of porous materials in catalysis, separation, and energy-related applications.

### 4.12. Critical Comparison with Literature: Reassessment of Classical IGC Results on MgY and NH_4_Y Zeolites

A rigorous evaluation of the present thermodynamic framework requires a direct comparison with earlier studies performed on similar systems using conventional inverse gas chromatography methodologies. In this context, the work of Bilgiç and Tümsek [38] provides a particularly relevant benchmark, as it reports dispersive and polar thermodynamic parameters for probe molecules adsorbed on MgY- and NH_4_Y-type zeolites.

The comparison presented in Tables S8 and S9 reveals that, for nonpolar probes such as n-alkanes, both approaches yield broadly comparable trends in dispersive enthalpy and entropy. This apparent agreement reflects the dominant role of London dispersion interactions, which are relatively insensitive to methodological details when probe–surface interactions remain weakly specific. However, even within this homologous series, systematic deviations become noticeable as molecular size increases, indicating that the classical treatment does not fully capture the temperature dependence of interaction geometry.

The situation changes markedly for polar molecules. For probes such as dichloromethane, chloroform, tetrahydrofuran, and acetone, the discrepancies between the two sets of results become substantial, particularly for NH_4_Y. As quantified in Table 10, the relative errors in dispersive enthalpy and entropy frequently exceed several hundred percent and, in some cases, approach or surpass one order of magnitude. These deviations are even more pronounced for the polar contributions (Table 11), where extreme discrepancies are observed, notably for benzene and acetone, with errors reaching several thousand percent. Such magnitudes clearly exceed any reasonable experimental uncertainty and point to intrinsic methodological limitations.

These differences originate from fundamental assumptions embedded in classical IGC analyses. In conventional treatments, the molecular surface area of probe molecules is considered constant and independent of both temperature and the nature of the adsorbing surface. The present results demonstrate that this assumption is not physically valid: the molecular surface area varies systematically with temperature and follows a quadratic dependence, reflecting the adaptive nature of adsorption geometry. Neglecting this effect directly propagates into the calculation of dispersive surface energies, leading to systematic underestimation or overestimation depending on the probe and surface considered.

A second critical limitation lies in the implicit assumption of a fixed intermolecular separation distance. In reality, the present work shows that the intermolecular distance evolves linearly with temperature, modifying the strength of London interactions through the $r^{-6}$ dependence of dispersion forces. Ignoring this variation results in an inaccurate representation of the interaction potential and consequently affects all derived thermodynamic parameters.

Equally important is the treatment of polar contributions. Classical methods typically rely on linear extrapolation procedures to separate dispersive and specific interactions, assuming that these contributions are independent. However, adsorption on zeolitic surfaces involves strong coupling between dispersive, electrostatic, and hydrogen-bonding interactions. The present results clearly show that this coupling leads to nonlinear behavior, which cannot be captured by linear separation schemes. This explains the large discrepancies observed for polar probes, particularly on NH_4_Y, where protonic and hydrogen-bonding effects are significant.

In contrast, the methodology developed in this work introduces a thermodynamically consistent framework that explicitly accounts for these effects. By incorporating temperature-dependent molecular surface area and intermolecular distance, and by employing a Hamaker-based description of dispersive interactions combined with a nonlinear five-parameter model for Lewis acid–base interactions, the present approach provides a unified and physically grounded interpretation of adsorption phenomena.

The improved consistency of the results, particularly for polar systems, demonstrates that the discrepancies observed with classical methods are not accidental but systematic. They arise from oversimplified assumptions that neglect the dynamic and coupled nature of adsorption at the molecular scale. In this respect, the present comparison not only validates the proposed methodology but also highlights the necessity of revisiting conventional interpretations of IGC data for complex materials.

From a broader perspective, this analysis underscores a key point: accurate surface characterization requires a framework in which structural, energetic, and electronic parameters are treated as interdependent and temperature-dependent variables. By establishing this connection, the present work moves beyond empirical descriptions and provides a deeper understanding of adsorption mechanisms, thereby offering a more reliable basis for the interpretation and prediction of surface properties in porous materials.

In this context, the present results not only resolve long-standing inconsistencies in the interpretation of IGC data but also establish a physically grounded and predictive framework for adsorption thermodynamics, thereby laying the foundation for the generalized approach and practical implications discussed in Section 5.

## 5. Conclusions

This work presents a comprehensive thermodynamic and molecular-level characterization of adsorption on MgY and NH_4_Y zeolites using inverse gas chromatography at infinite dilution combined with an advanced theoretical framework. The results demonstrate that adsorption is governed by a coupled interplay between dispersive, polar, and Lewis acid–base interactions, rather than by simple additive contributions.

From a quantitative standpoint, the London dispersive free energy exhibits a highly linear temperature dependence (R^2^ ≈ 1), while the corresponding surface energy decreases linearly with temperature, confirming the progressive weakening of dispersion forces. The intermolecular separation distance follows a linear law $r(T)=r_{0}+\alpha T$, with $\alpha_{eff}$ on the order of 10^−3^ Å·K^−1^, enabling the determination of intrinsic contact distances at 0 K. In addition, the molecular surface area of adsorbed probes is shown to vary quadratically with temperature (R^2^ > 0.999), demonstrating that the adsorption cross-section is not a fixed geometric parameter but a true thermodynamic quantity.

A major outcome of this study is the clear demonstration that classical IGC approaches, based on simplified assumptions such as fixed intermolecular distance and temperature-invariant surface properties, are insufficient to describe adsorption on complex surfaces. In contrast, the present methodology explicitly incorporates temperature-dependent structural and energetic parameters, providing a physically consistent description of adsorption phenomena.

The implementation of the five-parameter Lewis acid–base model further reveals that nonlinear donor–acceptor interactions, amphoteric coupling, and second-order effects are essential for accurately capturing adsorption energetics. Statistical analysis confirms the superiority of this model (highest R^2^, lowest RMSE, AIC, and BIC), highlighting the intrinsic complexity of acid–base interactions on heterogeneous zeolite surfaces.

The comparative analysis between MgY and NH_4_Y identifies two distinct adsorption regimes. MgY exhibits a structured and energetically coherent interaction field, characterized by strong dispersive interactions enhanced by Mg^2+^-induced polarization. In contrast, NH_4_Y displays a more flexible and heterogeneous adsorption environment, dominated by hydrogen bonding and proton-mediated interactions, leading to lower dispersive energies and more complex thermodynamic behavior.

Importantly, the Hamaker-based determination of the London dispersive surface energy establishes a direct connection between macroscopic thermodynamic quantities and microscopic electronic properties, thereby bridging the gap between experimental measurements and molecular-scale interactions.

Beyond the specific case of zeolites, this work introduces a general thermodynamic framework in which intermolecular distance, surface energy, and molecular surface area are treated as temperature-dependent variables. This paradigm shift provides a more realistic description of adsorption and opens new avenues for the interpretation of interfacial phenomena.

From a practical perspective, these findings offer valuable guidelines for the rational design of porous materials with tailored surface properties. The ability to quantitatively link adsorption energetics to electronic structure and molecular parameters provides a powerful tool for optimizing materials in catalysis, separation, environmental remediation, and energy applications.

Future work will focus on extending this framework to more complex systems, including multicomponent adsorption, confined fluids in hierarchical porous structures, and dynamic adsorption processes under non-equilibrium conditions. In addition, coupling this thermodynamic approach with molecular simulations and electronic structure calculations will further enhance its predictive capability and broaden its applicability.

## Figures and Tables

**Table 1:** Enthalpy–entropy compensation parameters for dispersive adsorption. Linear relationships between dispersive adsorption enthalpy $\left(-\Delta H_{a}^{d}\right)$ and entropy $\left(-\Delta S_{a}^{d}\right)$ for MgY and NH_4_Y zeolites, including the isokinetic temperature $T_{\mathrm{iso}}$ and the corresponding regression coefficients $R^{2}$.

Materials	Dispersive Adsorption	$T_{iso}$	R^2^
MgY	$\left(-\Delta H^{d}\right)$$\;=\;701.4\;(-\Delta S^{d})$ − 2.749	701.4	0.9975
NH_4_Y	$\left(-\Delta H^{d}\right)$$\;=\;534.6\;(-\Delta S^{d})$ + 12.114	534.6	0.9876

**Table 3:** Temperature-dependent intermolecular separation distances. Values of effective thermal expansion coefficients $\alpha_{\mathrm{eff}}$, extrapolated distances at 0 K $r_{0}$, and regression coefficients $R^{2}$ for probe molecules adsorbed on MgY and NH_4_Y zeolites.

Solvents	MgY			NH_4_Y		
	$\alpha_{eff}$(Å/K)	$r_{0}$(Å)	R^2^	$\alpha_{eff}$(Å/K)	$r_{0}$(Å)	R^2^
n-Pentane	0.0026	3.764	0.9985	0.0009	4.618	0.9998
n-Hexane	0.0025	3.776	0.9986	0.0014	4.379	0.9996
n-Heptane	0.0024	3.787	0.9986	0.0018	4.19	0.9993
n-Octane	0.0024	3.813	0.9987	0.002	4.104	0.9992
n-Nonane	0.0024	3.792	0.9986	0.0021	3.999	0.999
CCl_4_	0.0025	3.776	0.9986	0.0013	4.422	0.9996
Nitromethane	0.0027	3.736	0.9984	−0.0007	5.388	0.9998
CH_2_Cl_2_	0.0027	3.733	0.9984	−0.0009	5.444	0.9997
Chloroform	0.0026	3.758	0.9985	0.0005	4.797	0.9998
Diethyl ether	0.0026	3.757	0.9985	0.0005	4.809	0.9998
THF	0.0027	3.74	0.9984	−0.0004	5.23	0.9997
Ethyl acetate	0.0026	3.756	0.9985	0.0004	4.837	0.9998
Acetone	0.0028	3.708	0.9983	−0.0016	5.343	0.9994
Acetonitrile	0.003	3.659	0.9981	−0.003	5.748	0.9979
Toluene	0.0025	3.774	0.9986	0.0012	4.455	0.9996
Benzene	0.0021	3.644	0.9989	0.0009	4.645	0.9998
Methanol	0.0033	3.566	0.9976	−0.0055	6.582	0.9931
Ethanol	0.0029	3.662	0.9981	−0.0033	5.928	0.9977
Cyclohexane	0.0025	3.772	0.9986	0.0012	4.491	0.9996
Trichloroethylene	0.0026	3.762	0.9985	0.0008	4.688	0.9998
Tetrachloroethylene	0.0027	3.884	0.9985	0.0008	4.841	0.9998

**Table 4:** Linear approximation of molecular surface area. Coefficients of the molecular surface area $a_{X/S}(T)$ for probe molecules adsorbed on MgY and NH_4_Y, the thermal expansion coefficient $\lambda_{X/S}$, the surface area at 298.15 K, and regression coefficients $R^{2}$.

Solvents	MgY			NH_4_Y		
	$\lambda_{X/S}$	$a_{X/S}(298.15)$	R^2^	$\lambda_{X/S}$	$a_{X/S}(298.15)$	R^2^
n-Pentane	0.2162	40.52	0.9614	0.2896	30.26	0.9673
n-Hexane	0.1402	46.87	0.9774	0.2040	39.30	0.9809
n-Heptane	0.1119	51.95	0.9827	0.1670	48.54	0.9851
n-Octane	0.0953	56.99	0.985	0.1407	56.40	0.9871
n-Nonane	0.0801	61.57	0.9858	0.1198	64.40	0.9878
CCl_4_	0.0511	38.04	0.9848	0.1077	31.58	0.9893
Nitromethane	0.0862	24.66	0.972	0.1462	12.73	0.9794
CH_2_Cl_2_	0.0515	23.95	0.9798	0.1157	12.23	0.9861
Chloroform	0.0533	30.22	0.9826	0.1151	20.82	0.9878
Diethyl ether	0.2604	37.46	0.9499	0.3261	24.87	0.958
THF	0.0397	25.93	0.9827	0.1071	14.77	0.9889
Ethyl acetate	0.0256	33.95	0.9834	0.1003	23.26	0.9919
Acetone	0.0257	23.66	0.982	0.1704	17.51	0.9904
Acetonitrile	0.0520	16.73	0.9697	0.2395	11.40	0.9801
Toluene	−0.0016	36.61	0.7032	0.0564	30.33	0.9935
Benzene	0.0853	45.66	0.9866	0.102	23.74	0.9896
Methanol	−0.0042	11.87	0.9995	0.1666	7.21	0.9936
Ethanol	0.0079	17.86	0.9721	0.1617	11.55	0.9922
Cyclohexane	0.0574	37.10	0.9841	0.1176	29.88	0.9885
Trichloroethylene	0.001	39.09	0.3569	0.0798	28.86	0.9939
Tetrachloroethylene	−0.02	33.88	0.9999	0.0496	25.13	0.9947

**Table 5:** Quadratic model of molecular surface area. Quadratic coefficients $\lambda_{1}$ and $\lambda_{2}$ of the molecular surface area $a_{X/S}(T)$ for probe molecules adsorbed on MgY and NH_4_Y, including the extrapolated values at 298.15 K and regression coefficients $R^{2}$, demonstrating the non-linear thermodynamic behavior of adsorption cross-sections.

Solvents	MgY				NH_4_Y			
	$\lambda_{2}$	$\lambda_{1}$	$a_{X/S}(298.15)$	R^2^	$\lambda_{2}$	$\lambda_{1}$	$a_{X/S}(298.15)$	R^2^
n-Pentane	0.0021	1.2648	44.72	0.9987	0.0026	1.5331	33.85	0.9989
n-Hexane	0.0011	0.5945	48.96	0.9996	0.0014	0.7783	41.14	0.9997
n-Heptane	0.0007	0.4016	53.41	0.9998	0.001	0.542	48.93	0.9998
n-Octane	0.0006	0.3115	58.16	0.9998	0.0008	0.4169	57.92	0.9999
n-Nonane	0.0005	0.2529	62.51	0.9998	0.0007	0.3415	69.04	0.9999
CCl_4_	0.0003	0.1683	39.17	0.9998	0.0006	0.2801	36.51	0.9999
Nitromethane	0.0007	0.4166	27.33	0.9993	0.0011	0.5851	19.22	0.9996
CH_2_Cl_2_	0.0004	0.204	27.61	0.9997	0.0007	0.3588	15.23	0.9998
Chloroform	0.0004	0.1913	35.23	0.9997	0.0006	0.328	18.85	0.9999
Diethyl ether	0.0029	1.773	41.42	0.9976	0.0033	1.9997	27.90	0.9982
THF	0.0003	0.142	29.91	0.9997	0.0006	0.2858	19.06	0.9999
Ethyl acetate	0.0002	0.0892	37.40	0.9997	0.0005	0.2145	28.40	0.9999
Acetone	0.0002	0.0942	26.47	0.9997	0.0008	0.4122	15.90	0.9999
Acetonitrile	0.0005	0.2639	21.75	0.9992	0.0017	0.9373	15.61	0.9996
Toluene	5.0 × 10^−5^	0.0376	36.55	0.9802	0.0002	0.1025	28.28	1.0000
Benzene	0.0005	0.259	47.12	0.9999	0.0005	0.2611	22.86	0.9999
Methanol	3.0 × 10^−6^	0.006	11.92	0.9997	0.0007	0.2957	11.72	1.0000
Ethanol	7.0 × 10^−5^	0.0382	18.32	0.9993	0.0007	0.3349	11.80	0.9999
Cyclohexane	0.0004	0.1948	41.18	0.9997	0.0006	0.3212	28.44	0.9999
Trichloroethylene	6.0 × 10^−5^	0.0431	38.92	0.9646	0.0003	0.1373	28.44	1.0000
Tetrachloroethylene	5.0 × 10^−6^	0.0237	33.87	0.9999	0.0002	0.0757	27.26	1.0000

**Table 6:** The $\gamma_{s}^{d}(T)$ equations of zeolite surfaces, with the London dispersive surface entropy $\varepsilon_{s}^{d}$, the extrapolated London dispersive surface energy at 0 K $\gamma_{s}^{d}(T=0\;K)$, and the intrinsic maximum temperature $T_{Max}$.

Zeolite Surface	MgY	NH_4_Y
Equation $\gamma_{s}^{d}\left(T\right)$ (mJ/m^2^)	$\gamma_{s}^{d}(T)$ = −0.297 *T* + 189.48	$\gamma_{s}^{d}(T)$ = −0.251 *T* + 160.05
$\varepsilon_{s}^{d}\;=\;{d\gamma }_{s}^{d}/dT$ (mJ m^−2^ K^−1^)	−0.297	−0.251
$\gamma_{s}^{d}(T=0\;K)$ (mJ/m^2^)	189.48	160.05
$T_{Max}$ (K)	637.98	637.65
R^2^	0.9997	0.9997

**Table 7:** Statistical comparison of the different Lewis acid–base thermodynamic models applied to the adsorption data of the investigated carbon materials. The models include the classical two-parameter model (2-P), the three-parameter model (3-P), two variants of four-parameter models (4.2-P and 4.3-P), and the Hamieh five-parameter model (5-P). The quality of the fits is evaluated using the coefficient of determination ($R^{2}$), Akaike information criterion (*AIC*), Bayesian information criterion (*BIC*), and root mean square error (*RMSE*). Normalized statistical indicators ($\overset{ˇ}{R^{2}}$, $\overset{ˇ}{AIC}$, $\overset{ˇ}{BIC}$, $\overset{ˇ}{RMSE}$) are also reported and combined into a global score used to rank the performance of the different models for each carbon material.

Zeolite	Model	$R^{2}$	*AIC*	*BIC*	*RMSE*	$\overset{ˇ}{R^{2}}$	$\overset{ˇ}{AIC}$	$\overset{ˇ}{BIC}$	$\overset{ˇ}{RMSE}$	*Score*
MgY	2-P	0.9665	80.7879	82.7051	3.4975	0.0000	0.9481	0.8193	1.0000	0.0465
	3-P	0.9702	81.1385	83.6948	3.2975	0.1891	1.0000	1.0000	0.8402	0.1076
	4.2-P	0.9763	79.9432	83.1385	2.9418	0.4977	0.8230	0.8984	0.5561	0.3436
	4.3-P	0.9759	80.1819	83.3772	2.9670	0.4770	0.8584	0.9420	0.5762	0.3155
	5-P	0.9862	74.3836	78.2179	2.2458	1.0000	0.0000	0.0000	0.0000	1.0000
NH_4_Y	2-P	0.9693	98.7799	100.6970	6.6501	0.0000	0.6486	0.4458	1.0000	0.1811
	3-P	0.9700	100.4530	103.0090	6.5729	0.0419	0.8564	0.7578	0.9648	0.1009
	4.2-P	0.9810	96.0515	99.2468	5.2296	0.6921	0.3097	0.2501	0.3531	0.6943
	4.3-P	0.9717	101.6090	104.8040	6.3777	0.1455	1.0000	1.0000	0.8760	0.0830
	5-P	0.9862	93.5586	97.3929	4.4543	1.0000	0.0000	0.0000	0.0000	1.0000

**Table 8:** Optimal Lewis acid–base adsorption models selected for each carbon material based on the statistical comparison presented in Table 7. The best model corresponds to the one providing the most favorable compromise between goodness of fit and model complexity, as evaluated by the combined score derived from the normalized statistical indicators ($\overset{ˇ}{R^{2}}$, $\overset{ˇ}{AIC}$, $\overset{ˇ}{BIC}$, $\overset{ˇ}{RMSE}$). The corresponding statistical parameters ($R^{2}$, *AIC*, *BIC*, *RMSE*) are reported for the selected model.

Solid	Best Model	$R^{2}$	*AIC*	*BIC*	*RMSE*	*Score*
MgY	5-P	0.9862	74.3836	78.2179	0.9862	1.0000
NH_4_Y	5-P	0.9862	93.5586	97.3929	4.4543	1.0000

**Table 9:** Lewis acid–base thermodynamic parameters of the investigated zeolite materials obtained from the different adsorption models. The parameters include the Lewis acidity constant $K_{A}$, the Lewis basicity constant $K_{D}$, the acidity–basicity ratio $K_{D}/K_{A}$, and the total polarity parameter $K_{A}+K_{D}$. For the extended models, additional parameters are reported: the amphoteric coupling parameter K and the second-order curvature parameters $K_{2A}$ and $K_{2D}$, which account for nonlinear donor–acceptor interactions. Highlighted rows correspond to the parameters obtained from the statistically optimal models identified in Table 8. The highlighted lines below correspond to the best model statistically validated.

Zeolite	Model	$K_{A}$	$K_{D}$	$K_{D}/K_{A}$	$K_{A}+K_{D}$	K	$K_{2A}$	$K_{2D}$
MgY	2-P	0.1453	0.4162	2.8652	0.5614	0	0	0
	3-P	0.1556	0.4994	3.2104	0.6550	−0.00035	0	0
	4.2-P	0.1186	0.6067	5.1145	0.7253	0.00073	0	−0.0065
	4.3-P	−0.0859	0.5570	−6.4857	0.4711	−0.00058	0.0053	0
	5-P	−0.2250	0.7231	−3.2141	0.4981	0.00078	0.0073	−0.0088
NH_4_Y	2-P	0.2973	0.8146	2.7400	1.1119	0	0	0
	3-P	0.3062	0.8868	2.8956	1.1930	−0.00076	0	0
	4.2-P	0.2075	1.1734	5.6557	1.3809	0.00052	0	−0.0175
	4.3-P	0.0395	0.9504	24.0844	0.9899	−0.00115	0.0059	0
	5-P	−0.2879	1.3413	−4.6581	1.0533	0.00058	0.0105	−0.0208

**Table 10:** Relative errors (%) in the dispersive thermodynamic parameters—enthalpy (ΔH_a_^d^) and entropy (ΔS_a_^d^)—for probe molecules adsorbed on MgY and NH_4_Y zeolites, calculated by comparing values reported by Bilgiç and Tümsek [38] with those obtained in the present work. The results highlight the magnitude of deviations arising from classical IGC methodologies, particularly for polar probes, and emphasize the impact of neglecting temperature-dependent molecular surface area and intermolecular distance on the determination of dispersive interaction parameters.

Solvents	MgY	MgY	NH_4_Y	NH_4_Y
	$\mathrm{Error}\;\mathrm{on}\;\Delta H_{a}^{d}$	$\mathrm{Error}\;\mathrm{on}\;\Delta S_{a}^{d}$	$\mathrm{Error}\;\mathrm{on}\;\Delta H_{a}^{d}$	$\mathrm{Error}\;\mathrm{on}\;\Delta S_{a}^{d}$
n-Hexane	0.002	0.029	3.48	3.24
n-Heptane	5.82	6.45	11.24	18.42
n-Octane	13.79	15.83	4.32	4.69
n-Nonane	0.002	1.40	0.002	1.29
CH_2_Cl_2_	8.73	17.74	514.16	459.76
Chloroform	22.99	9.31	99.83	383.46
THF	11.96	10.02	294.70	846.05
Ethyl acetate	10.08	2.17	107.80	448.91
Acetone	7.17	37.36	676.46	67.95
Benzene	30.61	35.56	133.17	267.04
Cyclohexane	45.66	27.67	21.32	8.28

**Table 11:** Relative errors (%) in the polar thermodynamic parameters—enthalpy (ΔH_a_^p^) and entropy (ΔS_a_^p^)—for probe molecules adsorbed on MgY and NH_4_Y zeolites, obtained by comparison with literature values from Bilgiç and Tümsek [38]. The large discrepancies observed, especially for strongly interacting probes, illustrate the limitations of classical separation procedures for dispersive and specific contributions and demonstrate the necessity of a thermodynamically consistent framework incorporating nonlinear and coupled interaction effects.

Solvents	MgY	MgY	NH_4_Y	NH_4_Y
	$\mathrm{Error}\;\mathrm{on}\;\Delta H_{a}^{p}$	$\mathrm{Error}\;\mathrm{on}\;\Delta S_{a}^{p}$	$\mathrm{Error}\;\mathrm{on}\;\Delta H_{a}^{p}$	$\mathrm{Error}\;\mathrm{on}\;\Delta S_{a}^{p}$
CH_2_Cl_2_	14.6	47.1	76.9	89.8
Chloroform	52.5	28.3	62.8	74.9
THF	21.9	27.3	75.9	88.2
Ethyl acetate	32.8	12.9	72.6	87.5
Acetone	13.4	154.8	81.8	95.2
Benzene	3522.2	4383.3	653.3	1204.0

## Data Availability

All data generated or analyzed during this study are included in this published article and its Supplementary Information file.

## Supplementary Materials

The following supporting information can be downloaded at: https://www.mdpi.com/article/10.3390/molecules31101760/s1. Table S1. Variations of London dispersive free energy ${∆G}_{a}^{d}(T)$ (in kJ/mol) of MgY and NH_4_Y as a function of temperature. Table S2. Variations of polar free energy ${∆G}_{a}^{p}(T)$ (in kJ/mol) of MgY and NH_4_Y as a function of temperature. Table S3. Variations of the separation distance r(T) between solvents and zeolite surfaces as a function of temperature. Table S4. Thermodynamic parameters of London dispersive adsorption. Linear expressions of the dispersive free energy of adsorption, $\Delta G_{a}^{d}(T)$, together with the corresponding dispersive enthalpy $\Delta H_{a}^{d}$, entropy $\Delta S_{a}^{d}$, compensation temperature $T_{\mathrm{int}}$, and regression coefficient $R^{2}$ for probe molecules adsorbed on MgY and NH_4_Y zeolites. Table S5. Thermodynamic parameters of polar adsorption. Linear expressions of the polar free energy of adsorption, $\Delta G_{a}^{p}(T)$, together with the corresponding polar enthalpy $\Delta H_{a}^{p}$, entropy $\Delta S_{a}^{p}$, compensation temperature $T_{\mathrm{int}}$, and regression coefficient $R^{2}$ for probe molecules adsorbed on MgY and NH_4_Y zeolites. Table S6. Variations of the surface area (Å^2^) of adsorbed solvents on zeolite surfaces as a function temperature for MgY and NH_4_Y. Table S7. Values of London dispersive surface energy of zeolite surfaces as a function of temperature. Table S8. Comparison between the dispersive enthalpy $\Delta H_{a}^{d}$, entropy $\Delta S_{a}^{d}$, for probe molecules adsorbed on MgY and NH_4_Y zeolites. The values with * are relative to our work, however, the others are those of Bilgic and Tumsek (J. Chromatography A, 2007) [38]. Table S9. Comparison between the polar enthalpy $\Delta H_{a}^{p}$, entropy $\Delta S_{a}^{p}$, for probe molecules adsorbed on MgY and NH_4_Y zeolites. The values with * are relative to our work, however, the others are those of Bilgic and Tumsek (J. Chromatography A, 2007) [38].
