# Supplementary material for: Thermodynamic and Molecular Characterization of Adsorption on Zeolites: A Unified Framework Combining Inverse Gas Chromatography, Hamaker Theory, and Nonlinear Lewis Acid–Base Modeling
Source: Molecules. 2026 May 20;31(10):1760. doi: 10.3390/molecules31101760 (PMC13209460; doi:10.3390/molecules31101760)
Supplement: Supplementary file 1 [file molecules-31-01760-s001.zip › molecules-4291546-supplementary.pdf]

Supplementary Materials

Thermodynamic and Molecular Characterization of Adsorption on Zeolites: A Unified Framework Combining Inverse Gas Chromatography, Hamaker Theory, and Nonlinear Lewis Acid–Base Modeling

Tayssir Hamieh [a,b,c*], Mouhamad Rachini[c], Soumaya Hamieh[c], Mohammad Mahdi Assaf[c,d], Zeinab Hamie[c,e], Khaled Chawraba[c,f], Thibault Roques-Carmes[d], Joumana Toufaily[c]

[a] Faculty of Science and Engineering, Maastricht University, P.O. Box 616, 6200 MD Maastricht, The Netherlands, Email: t.hamieh@maastrichtuniversity.nl

[b] Institut de Science des Matériaux de Mulhouse, Université de Haute-Alsace, CNRS, IS2M UMR 7361, F-68100 Mulhouse, France

[c] Laboratory of Materials, Catalysis, Environment and Analytical Methods Laboratory (MCEMA), and LEDDAR Laboratory, Lebanese University, Hadath, Lebanon.

[d] Université de Lorraine, CNRS, LRGP, F-54000 Nancy, France.

[e] TIMR (Integrated Transformations of Renewable Matter), Royallieu Research Center, University of Technology de Compiegne, ESCOM, CS 60 319, CEDEX, 60-203 Compiegne, France.

[f] Department of Civil and Environmental Engineering, The Hong Kong Polytechnic University, Hung Hom, Kowloon, Hong Kong

[*] Correspondence: t.hamieh@maastrichtuniversity.nl.

Table S1. Variations of London dispersive free energy $\Delta G_a^d(T)$ (in kJ/mol) of MgY and NH_4Y as a function of temperature.

			**MgY**					
Solvents	313.15	323.15	333.15	343.15	353.15	363.15	373.15	383.15
n-Pentane	19.803	19.216	18.628	18.041	17.454	16.866	16.279	15.691
n-Hexane	23.698	23.009	22.319	21.630	20.941	20.252	19.563	18.873
n-Heptane	27.261	26.486	25.711	24.936	24.161	23.386	22.611	21.836
n-Octane	30.861	29.994	29.126	28.258	27.391	26.523	25.656	24.788
n-Nonane	34.206	33.235	32.264	31.294	30.323	29.353	28.382	27.411
CCl4	22.733	22.074	21.415	20.749	20.090	19.424	18.765	18.106
Nitromethane	15.087	14.627	14.168	13.704	13.244	12.781	12.321	11.862
CH2 Cl2	14.876	14.420	13.964	13.508	13.052	12.596	12.140	11.684
Chloroform	18.431	17.884	17.338	16.785	16.239	15.687	15.140	14.593
Diethyl ether	18.213	17.672	17.131	16.584	16.043	15.497	14.956	14.415
THF	15.653	15.179	14.705	14.226	13.752	13.273	12.799	12.324
Ethyl acetate	18.024	17.488	16.952	16.410	15.874	15.333	14.796	14.260
Acetone	12.232	11.847	11.462	11.074	10.689	10.300	9.916	9.531
Acetonitrile	9.300	8.992	8.683	8.372	8.064	7.753	7.445	7.136
Toluene	22.003	21.363	20.723	20.076	19.436	18.790	18.150	17.510
Benzene	28.622	27.883	27.143	26.404	25.664	24.925	24.185	23.446
Methanol	6.427	6.193	5.960	5.725	5.492	5.257	5.023	4.790
Ethanol	9.447	9.134	8.822	8.507	8.195	7.880	7.568	7.256
Cyclohexane	21.578	20.949	20.320	19.685	19.056	18.420	17.791	17.163
Trichloroethylene	19.266	18.698	18.129	17.555	16.986	16.412	15.844	15.275
Tetrachloroethylene	19.266	18.698	18.129	17.555	16.986	16.412	15.844	15.275
			**NH_4Y**					
Solvents	313.15	323.15	333.15	343.15	353.15	363.15	373.15	383.15
n-Pentane	14.611	14.458	14.304	14.151	13.998	13.845	13.692	13.539
n-Hexane	18.963	18.651	18.339	18.027	17.715	17.403	17.091	16.780
n-Heptane	23.917	23.431	22.946	22.460	21.975	21.489	21.004	20.518
n-Octane	28.439	27.796	27.152	26.508	25.864	25.220	24.577	23.933
n-Nonane	33.175	32.362	31.548	30.735	29.921	29.108	28.294	27.481

CCl4	17.950	17.677	17.404	17.131	16.858	16.585	16.312	16.033
Nitromethane	8.171	8.244	8.318	8.392	8.466	8.539	8.613	8.683
CH2Cl2	7.898	7.981	8.065	8.148	8.231	8.315	8.398	8.477
Chloroform	12.442	12.364	12.286	12.209	12.131	12.053	11.976	11.893
Diethyl ether	12.323	12.249	12.176	12.102	12.029	11.955	11.882	11.804
THF	9.028	9.072	9.115	9.158	9.202	9.245	9.288	9.327
Ethyl acetate	12.032	11.969	11.906	11.843	11.780	11.717	11.653	11.585
Acetone	9.587	9.788	9.989	10.190	10.390	10.591	10.792	10.989
Acetonitrile	7.697	8.036	8.375	8.713	9.052	9.391	9.729	10.065
Toluene	17.299	17.050	16.800	16.550	16.300	16.050	15.800	15.545
Benzene	14.244	14.102	13.961	13.819	13.678	13.536	13.395	13.248
Methanol	5.059	5.527	5.995	6.462	6.930	7.397	7.865	8.331
Ethanol	6.956	7.286	7.615	7.945	8.274	8.604	8.933	9.260
Cyclohexane	16.629	16.403	16.177	15.951	15.725	15.499	15.272	15.040
Trichloroethylene	13.688	13.566	13.445	13.323	13.201	13.079	12.957	12.830
Tetrachloroethylene	13.688	13.566	13.445	13.323	13.201	13.079	12.957	12.830

Table S2. Variations of polar free energy $\Delta G_a^p(T)$ (in kJ/mol) of MgY and NH_4Y as a function of temperature.

				**MgY**				
Solvents	313.15	323.15	333.15	343.15	353.15	363.15	373.15	383.15
CCl4	0.849	0.830	0.811	0.792	0.773	0.754	0.736	0.717
Nitromethane	6.825	6.683	6.541	6.400	6.258	6.116	5.974	5.832
CH2Cl2	12.055	11.883	11.711	11.539	11.367	11.195	11.023	10.851
Chloroform	9.937	9.755	9.572	9.395	9.213	9.036	8.854	8.672
Diethyl ether	11.484	11.302	11.120	10.938	10.755	10.573	10.391	10.209
THF	11.130	10.952	10.774	10.601	10.422	10.249	10.071	9.893
Ethyl acetate	7.797	7.702	7.607	7.517	7.422	7.332	7.237	7.141
Acetone	10.155	10.061	9.967	9.876	9.782	9.692	9.598	9.504
Acetonitrile	13.124	12.885	12.647	12.408	12.170	11.931	11.693	11.454
Toluene	3.184	3.128	3.072	3.016	2.960	2.904	2.848	2.792

Benzene	0.272	0.266	0.261	0.255	0.249	0.244	0.238	0.232
Methanol	25.944	25.429	24.914	24.400	23.885	23.371	22.856	22.342
Ethanol	22.930	22.493	22.055	21.618	21.180	20.742	20.305	19.867
Cyclohexane	1.100	0.773	0.645	0.544	0.526	0.505	0.468	0.411
Trichloroethylene	1.303	1.277	1.251	1.225	1.199	1.172	1.146	1.120
Tetrachloroethylene	1.786	1.751	1.716	1.681	1.646	1.611	1.576	1.541
**NH_4Y**								
Solvents	313.15	323.15	333.15	343.15	353.15	363.15	373.15	383.15
CCl4	1.838	1.799	1.760	1.721	1.682	1.643	1.604	1.565
CCl4	13.432	13.119	12.806	12.493	12.180	11.867	11.554	11.241
Nitromethane	16.362	15.752	15.142	14.532	13.922	13.312	12.702	12.096
CH2Cl2	11.168	10.769	10.369	9.970	9.570	9.171	8.771	8.377
Chloroform	15.326	14.800	14.273	13.747	13.220	12.694	12.167	11.641
Diethyl ether	13.884	13.374	12.863	12.353	11.843	11.333	10.823	10.317
THF	10.523	10.194	9.864	9.535	9.206	8.877	8.548	8.224
Ethyl acetate	15.849	15.285	14.721	14.158	13.594	13.030	12.467	11.907
Acetone	21.405	20.804	20.202	19.600	18.999	18.397	17.795	17.194
Acetonitrile	4.955	4.809	4.663	4.517	4.371	4.225	4.079	3.933
Toluene	0.539	0.526	0.514	0.502	0.489	0.477	0.464	0.452
Benzene	47.872	46.682	45.493	44.303	43.114	41.925	40.735	39.546
Methanol	40.099	39.048	37.997	36.946	35.894	34.843	33.792	32.741
Ethanol	4.491	4.473	4.455	4.437	4.419	4.401	4.383	4.365
Cyclohexane	2.446	2.387	2.327	2.267	2.207	2.148	2.088	2.028
Trichloroethylene	3.234	3.152	3.070	2.988	2.906	2.824	2.742	2.661

Table S3. Variations of the separation distance r(T) between solvents and zeolite surfaces as a function of temperature.

**MgY**								
Temperature (K)	313.15	323.15	333.15	343.15	353.15	363.15	373.15	383.15
n-Pentane	4.576	4.599	4.623	4.648	4.674	4.700	4.728	4.757

n-Hexane	4.568	4.590	4.614	4.638	4.663	4.689	4.716	4.744
n-Heptane	4.557	4.579	4.601	4.625	4.649	4.675	4.701	4.728
n-Octane	4.576	4.598	4.620	4.644	4.668	4.693	4.719	4.746
n-Nonane	4.562	4.583	4.606	4.630	4.654	4.679	4.706	4.733
CCl4	4.569	4.592	4.615	4.640	4.665	4.691	4.718	4.746
Nitromethane	4.576	4.600	4.624	4.650	4.677	4.705	4.733	4.763
CH2Cl2	4.577	4.601	4.626	4.651	4.678	4.706	4.735	4.765
Chloroform	4.573	4.596	4.619	4.644	4.670	4.697	4.725	4.754
Diethyl ether	4.573	4.596	4.620	4.645	4.670	4.698	4.725	4.755
THF	4.576	4.599	4.623	4.649	4.675	4.703	4.732	4.762
Ethyl acetate	4.573	4.596	4.620	4.645	4.671	4.698	4.726	4.755
Acetone	4.581	4.605	4.631	4.658	4.685	4.714	4.744	4.775
Acetonitrile	4.589	4.615	4.641	4.670	4.699	4.730	4.762	4.796
Toluene	4.570	4.592	4.616	4.640	4.665	4.692	4.719	4.747
Benzene	4.295	4.314	4.333	4.353	4.374	4.395	4.417	4.440
Methanol	4.603	4.631	4.661	4.693	4.725	4.760	4.796	4.834
Ethanol	4.588	4.614	4.641	4.669	4.698	4.729	4.761	4.794
Cyclohexane	4.570	4.593	4.616	4.641	4.666	4.692	4.720	4.748
Trichloroethylene	4.572	4.595	4.618	4.643	4.669	4.696	4.723	4.752
Tetrachloroethylene	4.720	4.744	4.768	4.794	4.820	4.848	4.877	4.907
				**NH_4Y**				
Solvents	313.15	323.15	333.15	343.15	353.15	363.15	373.15	383.15
n-Pentane	4.898	4.907	4.916	4.924	4.933	4.942	4.952	4.961
n-Hexane	4.824	4.837	4.851	4.865	4.879	4.893	4.908	4.923
n-Heptane	4.740	4.756	4.772	4.789	4.807	4.825	4.843	4.862
n-Octane	4.721	4.739	4.758	4.777	4.797	4.817	4.838	4.859
n-Nonane	4.667	4.686	4.706	4.726	4.748	4.769	4.792	4.815
CCl4	4.833	4.845	4.858	4.870	4.884	4.897	4.910	4.925
Nitromethane	5.155	5.147	5.139	5.132	5.124	5.117	5.110	5.103
CH2Cl2	5.172	5.163	5.154	5.145	5.137	5.128	5.120	5.112
Chloroform	4.964	4.969	4.975	4.980	4.985	4.991	4.996	5.002
Diethyl ether	4.968	4.973	4.978	4.983	4.988	4.993	4.998	5.004

THF	5.106	5.102	5.097	5.093	5.089	5.085	5.082	5.078
Ethyl acetate	4.978	4.982	4.987	4.991	4.995	5.000	5.004	5.009
Acetone	4.856	4.839	4.823	4.807	4.791	4.776	4.761	4.747
Acetonitrile	4.813	4.779	4.746	4.715	4.685	4.656	4.629	4.603
Toluene	4.845	4.856	4.868	4.880	4.893	4.905	4.918	4.932
Benzene	4.912	4.920	4.929	4.937	4.945	4.954	4.963	4.972
Methanol	4.872	4.801	4.736	4.677	4.623	4.573	4.527	4.484
Ethanol	4.912	4.874	4.838	4.804	4.772	4.741	4.711	4.683
Cyclohexane	4.858	4.869	4.880	4.891	4.903	4.915	4.927	4.940
Trichloroethylene	4.927	4.934	4.942	4.949	4.957	4.965	4.972	4.981
Tetrachloroethylene	5.088	5.095	5.103	5.111	5.118	5.126	5.134	5.143

Table S4. Thermodynamic parameters of London dispersive adsorption. Linear expressions of the dispersive free energy of adsorption, $\Delta G_a^d(T)$, together with the corresponding dispersive enthalpy ΔH_a^d, entropy ΔS_a^d, compensation temperature T_{int}, and regression coefficient $R^2$for probe molecules adsorbed on MgY and NH_4Y zeolites.

Solvents	MgY				
	$\Delta G_a^d(T)$	ΔS_a^d	ΔH_a^d	T_{int}	R^2
n-Pentane	y = -0.0587x + 38.198	58.7	38.198	650.7	1.0000
n-Hexane	y = -0.0689x + 45.281	68.9	45.281	657.2	1.0000
n-Heptane	y = -0.0775x + 51.53	77.5	51.53	664.9	1.0000
n-Octane	y = -0.0868x + 58.03	86.8	58.03	668.5	1.0000
n-Nonane	y = -0.0971x + 64.601	97.1	64.601	665.3	1.0000
CCl4	y = -0.0662x + 43.45	66.2	43.45	656.3	1.0000
Nitromethane	y = -0.0461x + 29.525	46.1	29.525	640.5	1.0000
CH2 Cl2	y = -0.0456x + 29.156	45.6	29.156	639.4	1.0000
Chloroform	y = -0.0549x + 35.613	54.9	35.613	648.7	1.0000
Diethyl ether	y = -0.0543x + 35.217	54.3	35.217	648.6	1.0000
THF	y = -0.0476x + 30.556	47.6	30.556	641.9	1.0000
Ethyl acetate	y = -0.0538x + 34.876	53.8	34.876	648.3	1.0000

Acetone	y = -0.0386x + 24.324	38.6	24.324	630.2	1.0000
Acetonitrile	y = -0.0309x + 18.986	30.9	18.986	614.4	1.0000
Toluene	y = -0.0642x + 42.119	64.2	42.119	656.1	1.0000
Benzene	y = -0.0739x + 51.779	73.9	51.779	700.7	1.0000
Methanol	y = -0.0234x + 13.753	23.4	13.753	587.7	1.0000
Ethanol	y = -0.0313x + 19.254	31.3	19.254	615.1	1.0000
Cyclohexane	y = -0.0631x + 41.347	63.1	41.347	655.3	1.0000
Trichloroethylene	y = -0.0571x + 37.135	57.1	37.135	650.4	1.0000
Tetrachloroethylene	y = -0.0571x + 37.135	57.1	37.135	650.4	1.0000
Solvents	NH4Y				
	$\Delta G_a^d(T)$	ΔS_a^d	ΔH_a^d	T_{int}	R^2
n-Pentane	y = -0.0153x + 19.406	0.0153	19.406	1268.4	1.0000
n-Hexane	y = -0.0312x + 28.731	0.0312	28.731	920.9	1.0000
n-Heptane	y = -0.0486x + 39.121	0.0486	39.121	805.0	1.0000
n-Octane	y = -0.0644x + 48.599	0.0644	48.599	754.6	1.0000
n-Nonane	y = -0.0813x + 58.649	0.0813	58.649	721.4	1.0000
CCl4	y = -0.0274x + 26.516	0.0274	26.516	967.7	1.0000
Nitromethane	y = -0.0073x + 5.8727	0.0073	5.8727	804.5	1.0000
CH2Cl2	y = -0.0083x + 5.2999	0.0083	5.2999	638.5	1.0000
Chloroform	y = -0.0078x + 14.888	0.0078	14.888	1908.7	0.9999
Diethyl ether	y = -0.0074x + 14.636	0.0074	14.636	1977.8	1.0000
THF	y = -0.0043x + 7.6844	0.0043	7.6844	1787.1	0.9998
Ethyl acetate	y = -0.0064x + 14.023	0.0064	14.023	2191.1	0.9999
Acetone	y = -0.02x + 3.3099	0.02	3.3099	165.5	1.0000
Acetonitrile	y = 0.0338x - 2.9001	-0.0338	-2.9001	85.8	1.0000
Toluene	y = -0.025x + 25.14	0.025	25.14	1005.6	1.0000
Benzene	y = -0.0142x + 18.69	0.0142	18.69	1316.2	1.0000
Methanol	y = 0.0467x - 9.5794	-0.0467	-9.5794	205.1	1.0000

Ethanol	y = 0.0329x - 3.3545	-0.0329	-3.3545	102.0	1.0000
Cyclohexane	y = -0.0227x + 23.728	0.0227	23.728	1045.3	1.0000
Trichloroethylene	y = -0.0122x + 17.518	0.0122	17.518	1435.9	1.0000
Tetrachloroethylene	y = -0.0122x + 17.518	0.0122	17.518	1435.9	1.0000

Table S5. Thermodynamic parameters of polar adsorption. Linear expressions of the polar free energy of adsorption, $\Delta G_a^p(T)$, together with the corresponding polar enthalpy ΔH_a^p, entropy ΔS_a^p, compensation temperature T_{int}, and regression coefficient $R^2$for probe molecules adsorbed on MgY and NH_4Y zeolites.

**Solvents**	**MgY**				
	$\boldsymbol{\Delta G_a^p(T)}$	$\boldsymbol{\Delta S_a^p}$	$\boldsymbol{\Delta H_a^p}$	$\boldsymbol{T_{int}}$	**R^2**
CCl4	y=-0.0019x+1.443	0.0019	1.443	761.3	1.0000
Nitromethane	y=-0.0142x+111.2268	0.0142	11.268	794.2	1.0000
CH2Cl2	y = -0.0172x + 17.441	0.0172	17.441	1014.0	1.0000
Chloroform	y = -0.018x + 15.585	0.0180	15.585	865.8	1.0000
Diethyl ether	y = -0.0182x + 17.190	0.0182	17.190	943.4	1.0000
THF	y = -0.0176x + 16.654	0.0176	16.654	946.3	1.0000
Ethyl acetate	y = -0.0093x + 10.717	0.0093	10.717	1152.4	1.0000
Acetone	y = -0.0093x + 13.055	0.0093	13.055	1403.8	1.0000
Acetonitrile	y = -0.0238x + 20.590	0.0238	20.590	863.5	1.0000
Toluene	y = -0.0056x + 4.937	0.0056	4.937	881.7	1.0000
Benzene	y = -0.0006x + 0.450	0.0006	0.450	791.8	1.0000
Methanol	y = -0.0515x + 42.056	0.0515	42.056	817.4	1.0000
Ethanol	y = -0.0438x + 36.633	0.0438	36.633	837.2	1.0000
Cyclohexane	y = -0.0081x + 3.4339	0.0081	3.434	423.9	1.0000
Trichloroethylene	y = -0.0026x + 2.123	0.0026	2.123	811.2	1.0000
Tetrachloroethylene	y = -0.0035x + 2.88	0.0035	2.880	824.3	1.0000
Solvents	NH_4Y				
	$\Delta G_a^p(T)$	ΔS_a^p	ΔH_a^p	T_{int}	R^2

CCl4	y = -0.0039x + 3.057	0.00389	3.05716	785.254	1.0000
Nitromethane	y = -0.0313x + 23.23	0.03129	23.2301	742.421	1.0000
CH2Cl2	y = -0.061x + 35.452	0.061	35.452	581.18	1.0000
Chloroform	y = -0.0399x + 23.662	0.0399	23.662	593.033	1.0000
Diethyl ether	y = -0.0527x + 31.814	0.05265	31.8139	604.24	1.0000
THF	y = -0.051x + 29.846	0.051	29.846	585.216	1.0000
Ethyl acetate	y = -0.0329x + 20.816	0.0329	20.816	632.705	1.0000
Acetone	y = -0.0563x + 33.489	0.0563	33.489	594.831	1.0000
Acetonitrile	y = -0.0602x + 40.246	0.06017	40.2462	668.925	1.0000
Toluene	y = -0.0146x + 9.526	0.0146	9.52559	652.621	1.0000
Benzene	y = -0.0013x + 0.929	0.00125	0.9293	745.437	1.0000
Methanol	y = -0.119x + 85.117	0.11894	85.1171	715.641	1.0000
Ethanol	y = -0.1051x + 73.019	0.10512	73.0191	694.595	1.0000
Cyclohexane	y = -0.0018x + 5.0549	0.0018	5.0549	2808.28	1.0000
Trichloroethylene	y = -0.006x +4.317	0.00598	4.31742	722.563	1.0000
Tetrachloroethylene	y = -0.00082x + 5.798	0.00819	5.79782	708.09	1.0000

Table S6. Variations of the surface area (Å^2) of adsorbed solvents on zeolite surfaces as a function temperature for MgY and NH_4Y.

				MgY				
Solvents	313.15	323.15	333.15	343.15	353.15	363.15	373.15	383.15
n-Pentane	45.05	46.252	47.652	49.305	51.285	53.704	56.729	60.631
n-Hexane	49.642	50.533	51.544	52.7	54.034	55.589	57.424	59.621
n-Heptane	54.102	54.853	55.695	56.644	57.722	58.954	60.376	62.032
n-Octane	58.807	59.465	60.197	61.017	61.941	62.986	64.179	65.552
n-Nonane	63.072	63.628	64.248	64.94	65.717	66.594	67.591	68.732
CCl4	39.011	39.367	39.763	40.195	40.695	41.247	41.893	42.634
Nitromethane	26.394	26.918	27.518	28.204	29.018	29.972	31.132	32.557
CH2 Cl2	24.944	25.278	25.656	26.086	26.579	27.149	27.814	28.6

Chloroform	31.236	31.597	32.001	32.447	32.965	33.545	34.226	35.018
Diethyl ether	43.111	44.454	46.041	47.93	50.259	53.16	56.941	62.062
THF	26.673	26.942	27.243	27.574	27.96	28.391	28.898	29.487
Ethyl acetate	34.444	34.62	34.818	35.03	35.282	35.557	35.885	36.263
Acetone	24.151	24.323	24.518	24.731	24.982	25.26	25.59	25.974
Acetonitrile	17.802	18.111	18.468	18.877	19.367	19.944	20.652	21.528
Toluene	36.606	36.569	36.538	36.502	36.486	36.469	36.478	36.505
Benzene	47.259	47.861	48.527	49.267	50.096	51.028	52.084	53.29
Methanol	11.819	11.778	11.737	11.692	11.652	11.607	11.568	11.531
Ethanol	18.02	18.068	18.124	18.185	18.263	18.348	18.457	18.586
Cyclohexane	38.19	38.585	39.027	39.51	40.07	40.692	41.419	42.258
Trichloroethylene	39.132	39.115	39.106	39.093	39.104	39.114	39.154	39.216
Tetrachloroethylene	33.592	33.397	33.2	32.991	32.794	32.586	32.393	32.206
**NH_4Y**								
Solvents	313.15	323.15	333.15	343.15	353.15	363.15	373.15	383.15
n-Pentane	36.196	37.899	39.854	42.126	44.806	48.026	51.985	57.002
n-Hexane	43.26	44.612	46.128	47.841	49.793	52.04	54.659	57.755
n-Heptane	51.69	52.849	54.136	55.572	57.187	59.014	61.102	63.51
n-Octane	59.017	60.017	61.12	62.345	63.712	65.246	66.981	68.96
n-Nonane	66.618	67.476	68.423	69.47	70.636	71.941	73.41	75.078
CCl4	33.545	34.333	35.197	36.147	37.198	38.367	39.675	41.136
Nitromethane	15.567	16.524	17.597	18.813	20.204	21.815	23.71	25.966
CH2 Cl2	14.422	15.238	16.139	17.139	18.26	19.524	20.963	22.611
Chloroform	22.962	23.79	24.7	25.706	26.825	28.079	29.495	31.095
Diethyl ether	31.765	33.559	35.642	38.098	41.048	44.678	49.286	55.373
THF	16.754	17.536	18.393	19.336	20.38	21.543	22.848	24.315
Ethyl acetate	25.041	25.806	26.635	27.536	28.52	29.6	30.791	32.1
Acetone	20.614	21.886	23.272	24.787	26.452	28.294	30.343	32.631
Acetonitrile	16.046	17.629	19.4	21.399	23.68	26.315	29.403	33.083
Toluene	31.343	31.786	32.262	32.775	33.332	33.936	34.598	35.311
Benzene	25.613	26.364	27.185	28.087	29.083	30.19	31.427	32.808
Methanol	10.133	11.448	12.858	14.376	16.016	17.795	19.733	21.851

Ethanol	14.451	15.695	17.04	18.497	20.085	21.822	23.733	25.842
Cyclohexane	32.051	32.903	33.84	34.872	36.018	37.297	38.735	40.351
Trichloroethylene	30.278	30.909	31.587	32.316	33.103	33.957	34.886	35.89
Tetrachloroethylene	25.991	26.39	26.816	27.272	27.762	28.29	28.862	29.474

Table S7. Values of London dispersive surface energy of zeolite surfaces as a function of temperature

T(K)	MgY zeolite	NH_4Y zeolite
313.15	96.36	81.25
323.15	93.53	78.85
333.15	90.66	76.42
343.15	87.75	73.96
353.15	84.79	71.46
363.15	81.77	68.91
373.15	78.70	66.31
383.15	75.56	63.64

Table S8. Comparison between the dispersive enthalpy ΔH_a^d, entropy ΔS_a^d, for probe molecules adsorbed on MgY and NH_4Y zeolites. The values with * are relative to our work, however, the others are those of Bilgic and Tumsek (J. Chromatography A, 2007).

Solvents	MgY	MgY	MgY	MgY	NH4Y	NH4Y	NH4Y	NH4Y
	ΔH_a^d	ΔH_a^d*	ΔS_a^d	ΔS_a^d*	ΔH_a^d	ΔH_a^d*	ΔS_a^d	ΔS_a^d*
n-Hexane	45.28	45.281	68.92	68.9	27.73	28.731	30.19	31.2
n-Heptane	48.53	51.53	72.5	77.5	43.52	39.121	57.55	48.6
n-Octane	50.03	58.03	73.06	86.8	46.5	48.599	61.38	64.4
n-Nonane	64.6	64.601	98.46	97.1	58.65	58.649	82.35	81.3
CH2 Cl2	31.7	29.156	37.51	45.6	32.55	5.2999	46.46	8.3
Chloroform	43.8	35.613	60.01	54.9	29.75	14.888	37.71	7.8
THF	34.21	30.556	42.83	47.6	30.33	7.6844	40.68	4.3
Ethyl acetate	38.39	34.876	52.63	53.8	29.14	14.023	35.13	6.4
Acetone	22.58	24.324	24.18	38.6	25.7	3.3099	33.59	20
Benzene	35.93	51.779	47.62	73.9	43.58	18.69	52.12	14.2
Cyclohexane	22.47	41.347	45.64	63.1	18.67	23.728	20.82	22.7

Table S9. Comparison between the polar enthalpy ΔH_a^p, entropy ΔS_a^p, for probe molecules adsorbed on MgY and NH_4Y zeolites. The values with * are relative to our work, however, the others are those of Bilgic and Tumsek (J. Chromatography A, 2007).

Solvents	MgY	MgY	MgY	MgY	NH4Y	NH4Y	NH4Y	NH4Y
	ΔH_a^p	ΔH_a^p*	ΔS_a^p	ΔS_a^p*	ΔH_a^p	ΔH_a^p*	ΔS_a^p	ΔS_a^p*
CH2 Cl2	14.9	17.44	25.3	17.2	8.2	35.45	6.2	61
Chloroform	7.4	15.59	12.9	18	8.8	23.66	10	39.9
THF	13	16.65	22.4	17.6	7.2	29.85	6	51
Ethyl acetate	7.2	10.72	10.5	9.3	5.7	20.82	4.1	32.9
Acetone	14.8	13.06	23.7	9.3	6.1	33.49	2.7	56.3
Benzene	16.3	0.45	26.9	0.6	7	0.929	16.3	1.25
Cyclohexane		3.434		8.1		5.055		1.8
